# Promiscuous *Coxiella burnetii* CD4 Epitope Clusters Associated With Human Recall Responses Are Candidates for a Novel T-Cell Targeted Multi-Epitope Q Fever Vaccine

**DOI:** 10.3389/fimmu.2019.00207

**Published:** 2019-02-15

**Authors:** Anja Scholzen, Guilhem Richard, Leonard Moise, Laurie A. Baeten, Patrick M. Reeves, William D. Martin, Timothy A. Brauns, Christine M. Boyle, Susan Raju Paul, Richard Bucala, Richard A. Bowen, Anja Garritsen, Anne S. De Groot, Ann E. Sluder, Mark C. Poznansky

**Affiliations:** ^1^InnatOss Laboratories B.V., Oss, Netherlands; ^2^EpiVax, Inc., Providence, RI, United States; ^3^Department of Cell and Molecular Biology, Institute for Immunology and Informatics, University of Rhode Island, Providence, RI, United States; ^4^Department of Biomedical Sciences, Colorado State University, Fort Collins, CO, United States; ^5^Vaccine and Immunotherapy Center, Massachusetts General Hospital, Boston, MA, United States; ^6^Department of Medicine, Yale University School of Medicine, New Haven, CT, United States

**Keywords:** *Coxiella burnetii*, Q fever, multi-epitope vaccine, immunoinformatics, IFNγ, T cell

## Abstract

*Coxiella burnetii*, the causative agent of Q fever, is a Gram-negative intracellular bacterium transmitted via aerosol. Regulatory approval of the Australian whole-cell vaccine Q-VAX® in the US and Europe is hindered by reactogenicity in previously exposed individuals. The aim of this study was to identify and rationally select *C. burnetii* epitopes for design of a safe, effective, and less reactogenic T-cell targeted human Q fever vaccine. Immunoinformatic methods were used to predict 65 HLA class I epitopes and 50 promiscuous HLA class II *C. burnetii* epitope clusters, which are conserved across strains of *C. burnetii*. HLA binding assays confirmed 89% of class I and 75% of class II predictions, and 11 HLA class II epitopes elicited IFNγ responses following heterologous DNA/DNA/peptide/peptide prime-boost immunizations of HLA-DR3 transgenic mice. Human immune responses to the predicted epitopes were characterized in individuals naturally exposed to *C. burnetii* during the 2007–2010 Dutch Q fever outbreak. Subjects were divided into three groups: controls with no immunological evidence of previous infection and individuals with responses to heat-killed *C. burnetii* in a whole blood IFNγ release assay (IGRA) who remained asymptomatic or who experienced clinical Q fever during the outbreak. Recall responses to *C. burnetii* epitopes were assessed by cultured IFNγ ELISpot. While HLA class I epitope responses were sparse in this cohort, we identified 21 HLA class II epitopes that recalled T-cell IFNγ responses in 10–28% of IGRA+ subjects. IGRA+ individuals with past asymptomatic and symptomatic *C. burnetii* infection showed a comparable response pattern and cumulative peptide response which correlated with IGRA responses. None of the peptides elicited reactogenicity in a *C. burnetii* exposure-primed guinea pig model. These data demonstrate that a substantial proportion of immunoinformatically identified HLA class II epitopes show long-lived immunoreactivity in naturally infected individuals, making them desirable candidates for a novel human multi-epitope Q fever vaccine.

## Introduction

Q fever is a zoonotic disease that is transmitted to humans predominantly by aerosol from infected ruminants such as goats, sheep, and cattle and has a global public health impact (1). Its causative agent, the small Gram-negative coccobacillus *Coxiella burnetii*, infects a wide range of vertebrate and invertebrate hosts, is very stable in the environment and highly contagious; it is estimated that a single inhaled organism can result in infection (2). As a result, *C. burnetii* is also considered to be a potential biothreat agent (3). Q fever is endemic in many countries worldwide, with outbreaks occurring mainly in occupational settings, including the livestock industry and deployed military personnel (1). The largest reported outbreak occurred in the Netherlands from 2007 to 2010 with an estimated 40,000 infections at the center of the epidemic area alone (4). Infection remains asymptomatic in an estimated 50–60% of individuals (1). Acute *C. burnetii* infection, when identified clinically and serologically, can be treated with antibiotics such as doxycycline. However, long-term complications of infection are common; 10–20% of patients with acute Q fever later develop Q fever fatigue syndrome, and 1–5% of (often asymptomatically) infected individuals progress to persistent infection known as chronic Q fever, manifesting as endocarditis, aneurysms or vascular infections in individuals with specific risk factors (1, 5). Therefore, a preventive Q fever vaccine is considered critical in occupational and biodefense settings (6).

The two currently available Q fever vaccine formulations, Q-VAX® for humans (licensed for use in Australia only) and COXEVAC® for ruminant animals such as goats (licensed in the European Union), are inactivated whole cell vaccines based on phase I *C. burnetii*. Although Q-VAX® is strongly protective (7, 8), reported side effects in previously exposed individuals, in particular at the site of injection, necessitate pre-vaccination screening and are a daunting hurdle to regulatory approval in the US and Europe (6, 9). Hence, there is a clear need for a less reactogenic and at least equally efficacious vaccine for occupational and biodefense purposes.

Subunit vaccines have the advantage over traditional whole cell vaccines that they are better tolerated and offer the opportunity for rational design, both in terms of the target antigens and the appropriate delivery or adjuvant system selected to elicit the desired immune response (10). While both humoral and cellular immune responses are elicited upon *C. burnetii* infection and administration of whole cell vaccines (11), antibodies alone are insufficient to resolve infection (12, 13). Results from studies in murine infection models suggest that T-cell responses, particularly Th1 responses, are critical for clearance of the bacteria (13–15). The Th1 cytokine IFNγ has been shown to restore phagosome maturation and facilitate intracellular killing of *C. burnetii* (16, 17). Accordingly, a proof of concept study showed that partial protection in C57BL/6 mice can be elicited by a vaccine comprising seven *C. burnetii* CD4 epitopes (18).

In this context, the objective of the Q-VaxCelerate consortium is to develop a non-reactogenic T-cell-targeted vaccine to prevent Q fever disease in humans (19). To rationally select epitopes for inclusion in such a vaccine, we set out to identify HLA class I and class II *C. burnetii* epitopes using a combination of immunoinformatic and experimental methods. A library of computationally predicted human T-cell epitopes derived from *C. burnetii* was assessed for human HLA binding *in vitro*, immunogenicity and reactogenicity were determined in animal models, and antigenicity was investigated in a cohort of individuals naturally exposed to *C. burnetii* during the 2007–2010 Dutch Q fever outbreak. Using this systematic approach, we successfully identified a set of epitopes that recalls long-term memory IFNγ T-cell responses in humans and thus represents a promising first step in the development of a T-cell based human multi-epitope Q fever vaccine.

## Materials and Methods

### Ethics Statement

Animal research protocols for studies with HLA-DR3 transgenic mice performed by EpiVax were reviewed and approved by TGA Sciences Incorporated Institutional Animal Care and Use Committee (P07-10R20-EV69, P07-10R20-EV71). Animal research protocols for guinea pig experiments were reviewed and approved by the Colorado State University Institutional Animal Care and Use Committee (14-5305A, 16-6844A). All animal experimental activities were conducted in full compliance with university, federal and international regulations and the standards of the DoD Animal Care and Use Review Office. Methods of euthanasia as described below were consistent with the recommendations of the Panel on Euthanasia of the American Veterinary Medical Association (AVMA).

The human study was carried out in accordance with the recommendations of the Medical Ethical Committee Brabant (Tilburg, Netherlands). All subjects gave written informed consent in accordance with the Declaration of Helsinki. The protocol was reviewed and approved by the Medical Ethical Committee Brabant (Tilburg, Netherlands, NL51305.028.15).

### Immunoinformatics

#### Sequence Collection

*C. burnetii* antigens used in epitope predictions were immune-dominant antigens that were recognized by sera from *C. burnetii*-exposed humans and demonstrated to stimulate CD4^+^ T-cell and/or antibody responses in mice, as reported in the literature (18, 20–29), and type IV secretion system (T4SS) effectors (21, 23, 30–37). Of 94 T4SS protein sequences retrieved from the UniProt database (38), 53 were selected for immunoinformatic analysis; 20 putative effectors lacking experimental support for secretion, eight hypothetical proteins from pseudogenes and 13 proteins with discontinued database records were rejected. An additional 40 sequences were retrieved from UniProt for the immune-dominant antigen set. All sequences were retrieved from the reference RSA 493/Nine Mile phase I *C. burnetii* strain. Six additional complete *C. burnetii* genomes available in 2015 were obtained from UniProt for homology analyses: (i) RSA 331/Henzerling II, (ii) CbuK_Q154, (iii) Namibia, (iv) MSU Goat Q177, (v) CbuG_Q212, and (vi) Dugway 5J108-111. Of note, genome sequences were completed for two *C. burnetii* strains associated with the Dutch Q fever outbreak (39) during the course of this work, and all but one of the selected epitopes (p69) were 100% conserved in the two Dutch strains.

#### Epitope Prediction

Sequences for all antigens from the reference Nine Mile strain were scored for binding potential against a panel of HLA class II and class I alleles using the EpiMatrix algorithm (40). This algorithm as well as the ClustiMer and JanusMatrix algorithms discussed below are part of the proprietary iVAX toolkit developed by EpiVax, which is available for use under license or by collaboration (http://epivax.com/immunogenicity-screening/ivax-web-based-vaccine-design). Epitopes which were 100% identical in the corresponding antigens from at least six of the seven genomes (HLA class I epitopes) or all seven genomes (HLA class II epitopes) were prioritized. Class II epitopes were identified for eight supertype HLA-DR alleles: DRB1^*^0101, DRB1^*^0301, DRB1^*^0401, DRB1^*^0701, DRB1^*^0801, DRB1^*^1101, DRB1^*^1301, and DRB1^*^1501. For class I epitopes, selections were made based on predictions for six HLA-A and HLA-B supertype alleles: A^*^0101, A^*^0201, A^*^0301, A^*^2402, B^*^0702, B^*^4403. Each set of supertype alleles covers >95% of the human population worldwide (41, 42). EpiMatrix assigns a score for each peptide/allele pair on a normalized Z-scale. Peptides with Z-Scores ≥1.64 are considered hits and have a significant likelihood of binding to HLA molecules. These sequences represent the top 5% of any randomly generated set of 9-mer peptides (43).

Regions of high class II epitope density per antigen were identified using the ClustiMer algorithm for selection of vaccine candidates with increased breadth of reactivity (40). ClustiMer searches for contiguous segments of 15–30 amino acids with elevated class II HLA binding promiscuity. Epitope density within a cluster is estimated by EpiMatrix Cluster Scores, where scores of 10 and above are considered elevated. Peptide sequences with elevated EpiMatrix Cluster Scores usually contain class II HLA binding motifs to most, if not all, HLA-DR supertype alleles. MHC Class II epitope clusters were further filtered to remove sequences derived from signal sequences and transmembrane domains to avoid peptide synthesis and solubility complications. For class I peptides, epitopes with Z-Scores in the top 1% of a normal distribution (Z-Score ≥2.32) were prioritized. A greater weight was given to the top 1% EpiMatrix scoring peptides per HLA class I supertype that were also predicted to bind to at least one additional HLA class I supertype.

#### Homology Analysis

To eliminate peptide candidates unlikely to stimulate effector T-cell responses due to potential cross-reactivity with T-cells previously trained on host or commensal antigens, predicted HLA class I and class II *Coxiella* sequences were screened for T-cell receptor-face homology against host and commensal proteins using the JanusMatrix algorithm (44). Databases comprising the UniProt reviewed human proteome (http://www.uniprot.org/proteomes/UP000005640) and Human Microbiome Project gut commensal proteomes were used. Epitopes with low human or human microbiome JanusMatrix homology scores were prioritized. The JanusMatrix human homology score cutoff was 2, for both HLA class I and II, and the microbiome cutoff was 25 for HLA class I and 37 for HLA class II. JanusMatrix cutoffs are defined such that only 5% of randomly generated HLA ligands exceed the thresholds.

### Peptide Synthesis

Five milligrams of peptide for each epitope selected as defined above were prepared by Fmoc solid phase peptide synthesis (Twentyfirst Century Biochemicals, Marlborough, MA, United States) and HPLC purified to >90% purity (peak area). The peptide mass was verified by ESI-MS (QSTAR XL Pro, Qo-TOF) and the sequence of each peptide was verified by tandem mass spectrometry (CID MSMS).

### HLA Binding Assays

#### HLA Class II Binding Assay

A competition-based assay based on Steere et al. was employed (45). In 96-well plates, non-biotinylated test peptides over a three-log concentration range competed for binding to 50 nM soluble class II molecules (Benaroya Institute, Seattle, WA) against a biotinylated standard peptide at a fixed concentration (25 nM) at 37°C for 24 h to reach equilibrium. Class II molecules were then captured on ELISA plates using pan anti-class II antibodies (clone L243, anti-HLA-DR; Biolegend). Plates were washed and incubated with DELFIA Europium-labeled streptavidin (Perkin Elmer) for 1 h at room temperature. DELFIA Europium activation buffer (Perkin Elmer) was added to develop the plates for 15–20 min at room temperature before data acquisition on a Time Resolved Fluorescence (TRF) plate reader. All assays were performed in triplicate. Binding assays were performed for 8 alleles: DRB1^*^0101, DRB1^*^0301, DRB1^*^0401, DRB1^*^0701, DRB1^*^0801, DRB1^*^1101, DRB1^*^1301, and DRB1^*^1501. Dose-dependent binding data were fitted by non-linear regression to calculate IC_50_ values for each peptide-HLA allele pair (GraphPad Prism Version 7). Peptide binding affinity was classified according to the following: very high (IC_50_ < 0.1 μM), high (IC_50_ 0.1–1 μM), moderate (IC_50_ 1–10 μM) and low affinity (IC_50_ 10–100 μM). Epitopes with IC_50_ values too high to accurately measure under binding conditions tested (>100 μM) or with no dose-dependent responses were considered non-binders.

#### HLA Class I Binding Assay

A competition-based assay based on fluorescence polarization, where reference fluorescent-labeled peptide is incubated with soluble HLA with or without competitor test peptides, was performed according to Buchli et al. (46). Peptide binding over a three-log concentration range was assessed for the A^*^0101, A^*^0201, A^*^0301, A^*^2402, B^*^0702, and B^*^4402 alleles. The latter was used to test B^*^4403 predictions. A dose response curve fit was obtained by non-linear regression to calculate IC_50_ values for each peptide-HLA allele pair. Binding strength was classified according to IC_50_ value: high (<5 μM), moderate (5–50 μM), low (50–1,000 μM) or no affinity (>1,000 μM or no dose-dependent response).

### Assessment of Class II Epitope Immunogenicity in tgHLA-DR3 Mice

#### Multi-Epitope DNA Vaccine Engineering

Multi-epitope DNA vaccines were engineered by concatenating epitope sequences to form five multi-epitope genes, each containing 10 HLA class II epitopes. To avoid production of neo-epitopes at epitope junctions, the VaxCAD algorithm was used to arrange epitopes in an order that diminishes potential junctional immunogenicity (40). Where re-ordering by VaxCAD did not sufficiently reduce potential junctional immunogenicity, Gly-Pro-Gly-Pro-Gly spacer sequences were engineered between epitopes to further remove junctional epitopes. Genes were synthesized by GeneArt and subcloned at pre-determined flanking restriction sites downstream of the tissue plasminogen activator leader sequence in pNTC8682-eRNA41H (Nature Technology Corporation), a DNA vaccine vector that accommodates FDA recommendations for construction of plasmid DNA vaccines.

#### Plasmid DNA Vaccine Production

High purity plasmids for immunization were prepared by Nature Technology Corporation, Inc. at research grade. Each plasmid underwent quality control testing including spectrophotometric concentration and A260/A280 ratio determination (1.97), restriction digest analysis to assure the presence of the multi-epitope genes, agarose gel electrophoresis determination of residual host RNA and DNA (none detected), and quantitative endotoxin testing (<2.0 EU/mg).

#### Peptide Vaccine Preparation

Peptides produced by twentyfirst Century Biochemicals corresponding to epitopes in the DNA vaccines were formulated in incomplete Freund's adjuvant (IFA) with 10 μg each of immunostimulatory CpG oligodeoxynucleotide 1826 (5′-TCCATGACGTTCCTGACGTT-3′), muramyl dipeptide (MDP) and CL097 (InvivoGen).

#### Mice

HLA-DR3 transgenic mice were obtained from Dr. Chella David (Mayo Medical School) under commercial license to EpiVax. The mice express the HLA-DRA and DRB1^*^0301 genes on a B.10-Ab0 mouse class II-negative background (47).

#### Vaccinations

Vaccine and placebo-treated mice (*n* = 3/group) were all female and 6–8 weeks old at the start of heterologous DNA/DNA/peptide/peptide prime-boost immunizations. Epitopes were arranged into five groups of 10 epitopes in generally descending order of EpiMatrix epitope cluster score as follows: Group 1 (p9, p12, p14, p22, p27, p28, p31, p32, p45, p49), group 2 (p3, p7, p8, p15, p16, p38, p39, p42, p44, p46), group 3 (p4, p10, p19, p20, p21, p23, p25, p34, p40, p48), group 4 (p1, p2, p5, p6, p11, p13, p30, p36, p41, p47), group 5 (p17, p18, p24, p26, p29, p33, p35, p37, p43, p50). DR3 epitope scores correlated with cluster scores across the groups. DNA-prime vaccine was administered to mice intramuscularly by electroporation using the Ichor Medical Systems TriGrid platform with 20 μL of 10 μg naked plasmid DNA in sterile PBS injected into the quadriceps muscle. Mice received the DNA vaccine twice spaced by a 2-week interval. Two weeks later, they were boosted twice with peptide vaccine at a 2-week interval. For peptide immunizations, each mouse was administered 100 μl IFA emulsion (50 μg peptide) subcutaneously by needle stick injection. Four weeks after the final vaccination, tgHLA-DR3 mice were euthanized by administration of ketamine/xylazine intraperitoneally at 4–5 times the anesthetic dose (ketamine 80–100 mg/kg; xylazine 8–10 mg/kg).

#### Ex vivo ELISpot Assay in Mouse Splenocytes

The frequency of epitope-specific splenocytes was determined by IFNγ ELISpot assay using the colorimetric Mabtech IFNγ ELISpot Kit with pre-coated plates according to the manufacturer's protocol. Washed splenocytes in RPMI 1640 (Gibco) supplemented with 10% fetal calf serum (FCS, Atlanta Biologicals) were added at 2.5 × 10^5^ cells per well. Individual peptides were added at 10 μg/ml in triplicate wells. Peptide pools were added at 10 μg/ml, equating to 1 μg/ml per peptide. Triplicate wells were stimulated with 2 μg/ml Concanavalin A (ConA; Sigma Aldrich) as a positive control, and six replicate wells with medium containing 0.02% DMSO were used for background determination. Raw spot counts were recorded by ZellNet Consulting, Inc. using a Zeiss high-resolution automated ELISpot reader system and companion KS ELISpot software. Results were calculated as the average number of spots in the peptide wells, adjusted to spots per one million cells. A response in immunized mice was considered positive if the number of averaged spots was (i) at least twice as high as background (stimulation index ≥2), (ii) >50 spot forming cells above background per million splenocytes (1 response per 20,000 cells), and (iii) statistically different (*p* < 0.05) from that of mock immunized mice by Student's *t*-test.

### Human Study Cohort

Q fever exposed individuals were recruited from a cohort characterized in a previous large Q fever study conducted in the village of Herpen, the Netherlands (48), which experienced a high incidence of *C. burnetii* infection during the 2007–2010 Q fever outbreak (49). All subjects had been tested using a Q fever interferon-γ release assay (IGRA, Q-detect™) assay of cellular immunity during a previous study in which 80% of the adult population of Herpen were screened for Q fever (48). Individuals were invited to participate in the current study following preselection based on clinical history (disease, comorbidities, and medication) as well as IGRA and serological data generated during the previous study (48). To maximize the potential to detect *C. burnetii* epitope-specific T-cells, preference was given to donors with strong responses to whole heat-killed *C. burnetii* in the IGRA and without potentially confounding immune disorders. In addition, five individuals with known past symptomatic Q fever consented to participate. In total, 143 participants provided written informed consent. IGRA responses were re-assessed upon inclusion in October 2015. Volunteers who had no history of Q fever disease (48) and scored negative by immunofluorescence assay (50) as well as by IGRA in spring 2014 and upon inclusion into the present study in autumn 2015 were allocated to control group A (*n* = 26). Seven volunteers that were IGRA positive in 2014 but did not meet positive scoring thresholds anymore 1.5 years later upon inclusion into the present study were excluded from further analysis. The remaining 110 volunteers that were positive by IGRA in both 2014 and 2015 were subdivided based on past Q fever disease [either registered (notified) in the national surveillance system, or self-reported] into groups B (asymptomatic, *n* = 73) and C (symptomatic, *n* = 37).

### Whole Blood IFNγ Release Assay (Q-Detect™)

Whole lithium-heparin anti-coagulated blood was stimulated with *C. burnetii* antigen (heat killed Cb02629, Wageningen Bioveterinay Research, lot 14VRIM014) in 96-well polypropylene plates (Greiner BioOne) by adding 180 μl blood to 20 μl *C. burnetii* antigen diluted in phenol red-free RPMI supplemented with Glutamax (2 mM), Gentamycin (5 μg/ml) and sodium pyruvate (1 mM, all ThermoFisher Scientific). A 1.5 % (v/v, final concentration) solution of PHA-M (ThermoFisher Scientific), was added to separate wells as a positive control. Medium only was added to the negative control wells. All stimulations were performed in duplicate. After 22 ± 1 h whole blood cultures were re-suspended and IFNγ concentrations were assessed in whole blood by ELISA, using the IFNγ Pelipair protocol (Sanquin) with minor modifications. The upper detection limit of IGRA under these conditions is 1,050 pg/ml. A subject was scored positive by IGRA if the *C. burnetii*-induced IFNγ production was ≥16 pg/ml above background and the ratio of the logarithmic value of background-subtracted *C. burnetii* and PHA responses {(log[*C. burnetii*]–log[neg control])/(log[PHA]-log[neg control])} was ≥0.4.

### HLA Typing of Human Subjects

HLA typing was performed at the HLA laboratory at the Laboratory of Translational Immunology at the UMC Utrecht, the Netherlands. Genomic DNA was isolated from EDTA anti-coagulated blood within 48 h upon collection using the MagNA Pure Compact system (Roche Diagnostics). The DNA samples were typed for HLA-A, -B (5′-UTR-3′UTR), and -DRB1 (exon 2–3′-UTR) by Next Generation Sequencing (NGS). Firstly, HLA target sequences were generated by long-range PCR using the Qiagen LongRange PCR kit and HLA-A, -B, and -DRB1 primers as described previously (51). Library preparation was performed using the GenDX NGSgo®-LibrX and NGSgo®-IndX kits following the manufacturers' recommendations (GenDX). Pooled samples were sequenced on an Illumina MiSeq by 2 × 250 paired end reading using the MiSeq reagent kit v2 (500 cycles). Sequences were analyzed with the NGSEngine software (GenDX). The resulting HLA-A, HLA-B, and HLA-DRB1 alleles were assigned to supertype families as defined by (42, 52) and/or based on homology of the HLA binding pockets. For determining allelic frequencies, donors homozygous for a given HLA allele were counted once.

### Cultured ELISpot Assay Screening of Peptides in Human PBMCs

A combination of antigen-specific T-cell expansion culture and enzyme-linked immune spot assay (cultured ELISpot) was chosen as the primary assay for peptide screening, to achieve high sensitivity for detecting low frequency antigen-specific T-cell responses to *C. burnetii* peptides and facilitate detection of central memory T-cells (53). The protocol was adapted from Subbramanian et al. (54) and optimized at Innatoss using two reference peptide pools of HLA class I and class II peptides of CEF (Cytomegalovirus, Epstein-Barr virus, and influenza; JPT Peptide Technologies). A comparison of three media showed that RPMI medium supplemented with 10% FCS (HyClone) as a blocking and culture medium gave the best signal to background ratio in this ELISpot assay.

#### Antigen-Specific Expansion

PBMCs were isolated from lithium-heparin anti-coagulated blood using Leukosep tubes prefilled with Ficoll (Greiner BioOne) according to the manufacturer's recommendations. Isolated PBMCs were used for antigen-specific expansion cultures at 5 × 10^6^ cells per well in 48-well flat bottom plates (Corning) in 150 μl complete RPMI (phenol red-free RPMI supplemented with Glutamax (2 mM), Gentamycin (5 μg/ml), sodium pyruvate (1 mM, all Thermo Fisher Scientific) and 10% fetal bovine serum (HyClone). Antigen-specific expansion was performed using stimulation with pools of 10 peptides each (final concentration 2 μg/ml per peptide, 0.2% DMSO). On day 3 and 6 of culture, medium was refreshed by addition of an equal volume of complete RPMI with recombinant human IL-2 (Immunotools, final concentration 20 units/ml). On day 8, cells were harvested, counted and individual peptide responses assessed by ELISpot.

#### ELISpot Assay

MultiScreen IP filter plates (Merck Millipore) were pre-wetted with 35% ethanol, coated overnight with IFNγ capture antibody at the recommended concentration (Diaclone) in DPBS (Thermo Fisher Scientific) and blocked for at least 60 min with complete RPMI prior to addition of cells for re-stimulation. For each expansion culture, recovered cells were evenly distributed for peptide re-stimulation, negative and positive controls. Based on cell availability, a median of 38,000 cells per expansion pool (interquartile range (IQR) 27,000–52,000) were plated per replicate well. Assay wells were re-stimulated with each of the 10 respective individual peptides in quadruplicate (final concentration 2 μg/ml per peptide, 0.02% DMSO). Control wells were stimulated with either medium only (containing 0.02% DMSO; eight replicates) or with *Staphylococcus* enterotoxin B (SEB, final concentration 1 μg/ml, Sigma Aldrich; quadruplicate assays). After 20 h incubation at 37°C, plates were washed, and sequentially incubated with a biotinylated anti-human IFNγ detection antibody and streptavidin-alkaline phosphatase conjugate in DPBS/0.5% FCS and developed using BCIP-NBT (all Diaclone) according to the manufacturer's recommendation. ELISpot plates were dried overnight, scanned on an AID Classic reader system and analyzed using the AID ELISpot software v7.0 (both AID Diagnostika GmbH). Spot forming units were counted using identical settings for all plates and all counts were reviewed and adjusted manually only where necessary to remove artifacts.

#### Data Analysis

To account for variation in background responses between expansion cultures and donors following cytokine-assisted T-cell expansion and to decrease the likelihood of detecting false positive responses in plates with either high background (when only considering an absolute difference to background) or low background (when only considering a fold difference above background), three combined threshold criteria were applied. Peptide re-stimulation responses were defined as positive when they were (i) significantly higher than spot counts in matched negative control wells from the same expansion culture by one-way ANOVA with Holm-Šídák multiple comparison correction *post-hoc* test, reached (ii) a stimulation index of at least 2 above the matched negative control wells, and (iii) an absolute cut-off of >10 SFU/well.

### Guinea Pig Reactogenicity Against Class I and II Peptides

Female Dunkin-Hartley guinea pigs were sensitized by intranasal inoculation with 10^6^ genome equivalents of *C. burnetii* Nine Mile strain or saline in 100 μL volume, as described previously (55). Peptides were tested by intradermal challenge, delivered at day 42 post sensitization. HLA class II and I peptides were administered in pools of five, with each peptide being tested twice in two different pool preparations. Peptide pools were created with 2 μg of each peptide (10 μg peptide per pool in total) in 100 μL saline with 1% DMSO. Challenge with 2 μg COXEVAC® whole cell vaccine (Ceva Sante Animale, Libourne, France) was used as a positive control; negative controls consisted of saline or 1% DMSO injections. On day 7, animals were anesthetized (ketamine 40 mg/kg and xylazine 5 mg/kg, i.p.) and euthanized with beuthanasia (i.p.). Gross reactions were monitored daily and skin biopsies obtained at day 7 post-challenge were fixed, sectioned, and stained with hematoxylin and eosin. Histological reactions were scored by an experimenter blinded to the treatment group, using the criteria previously described (55). Briefly, a score of 0 indicates no inflammation, 1 indicates localized macrophage dominated inflammation, 2 macrophage dominated inflammation with limited tissue infiltrations, 3 lymphocytic inflammatory infiltrates extending into the deep dermis, 4 edema and increased pyogranulomatous inflammation extending deep into the subcutis, and 5 widespread pyogranulomatous inflammation including necrosis.

## Results

### *In silico* Identification of Predicted *C. burnetii* T-Cell Epitopes

Two *C. burnetii* antigen sets were selected as the basis for immunoinformatic identification of HLA class I and II T-cell epitopes. The first set (for HLA class I epitope prediction) was comprised of 53 published substrates of the type IV secretion system (T4SS), which are translocated from *C. burnetii* to the host cytoplasm where they are expected to enter the class I antigen processing pathway and trigger CD8^+^ T cell responses (21, 23, 30–37). The second set (for both HLA class I and II epitope prediction) covered 40 sero-reactive *C. burnetii* antigens based on antibody responses in humans and mice, as well as evidence of processing and presentation to stimulate murine CD4^+^ T-cells (18, 26, 28, 29, 56). Using the EpiMatrix algorithm, 8,643 putative 9- and 10-mer T-cell epitopes predicted to bind to at least one of six HLA class I supertype alleles and 282 promiscuous epitope clusters, spanning 14–25 amino acids, predicted to bind HLA class II alleles covering >90% of the world-wide human population, were identified from the reference *C. burnetii* Nine Mile strain (Table 1).

**Table 1:** Summary of *in silico* HLA class II and I epitope identification.

	**HLA class II**	**HLA class I**	
	**Sero-reactive**	**T4SS substrates**	**Sero-reactive**
Source antigens	40	53	40
Epitopes	282	8,643	5,100
Conserved across strains	188	3,971	4,578
High Scoring for HLA binding^a^	153	1,710	1,945
Different from human^b^	98	1,511	1,558
Without synthesis issues	81	1,108	1,163
Selected for immunogenicity tests^c^	50	30	35

a Evaluated by EpiMatrix.

b Evaluated by JanusMatrix.

c *No source antigen represented by more than two epitopes; five epitopes per antigen set for each of the 6 major HLA class I supertypes*.

The derived HLA class I epitopes and HLA class II promiscuous epitope clusters were then filtered to focus on sequences that (i) are conserved with other *C. burnetii* strains; (ii) have very high likelihood of binding human HLA alleles; (iii) exhibit low potential for cross-reactivity with peptides derived from the human proteome or microbiome based on the JanusMatrix algorithm (44); and (iv) do not present obvious issues for peptide synthesis or stability. Finally, 50 HLA class II epitope clusters (Table 2) and 65 HLA class I epitopes (Table 3) were selected for immune reactivity screening such that no source antigen was represented more than twice. Five epitopes were selected for each of the six HLA class I supertypes and for each antigen set (T4SS substrates and sero-reactive antigens), if possible giving preference to HLA class I ligands predicted to bind to at least two class I supertype alleles. An additional five HLA class I epitopes were specifically selected from the immunodominant *C. burnetii* antigen com1 (28, 56).

**Table 2:** HLA class II epitopes selected for immune reactivity screening.

**ID**	**Epitope**	**Source antigen**			**Predicted HLA II restrictions**
		**CBU code**	**UniProt ID**	**Gene name**	
p1	SEQITLQTAEKVGLNVA	CBU_1910	H7C7D7	com1	DRB1*0101, DRB1*0301, DRB1*0401, DRB1*0701, DRB1*1101, DRB1*1301, DRB1*1501
p2	TPTFVIGNKALTKFGF	CBU_1910	H7C7D7	com1	DRB1*0101, DRB1*0301, DRB1*0401, DRB1*0801, DRB1*1101, DRB1*1301, DRB1*1501
p3	KDDILEAVANMSVMDV	CBU_0229	P0C8S3	rplL	DRB1*0101, DRB1*0301, DRB1*0401, DRB1*0701, DRB1*0801, DRB1*1101, DRB1*1301, DRB1*1501
p4	KIGVIKAIRTITGLGLKEA	CBU_0229	P0C8S3	rplL	DRB1*0101, DRB1*0401, DRB1*0701, DRB1*0801, DRB1*1101, DRB1*1301, DRB1*1501
p5	LAQYRELEAFSQFAS	CBU_1943	Q83AF7	atpA	DRB1*0101, DRB1*0401, DRB1*0701, DRB1*0801, DRB1*1101, DRB1*1501
p6	SHEVLHAMSRGVEVLA	CBU_1718	P19421	groL	DRB1*0101, DRB1*0301, DRB1*0401, DRB1*0701, DRB1*0801, DRB1*1101, DRB1*1501
p7	SRAFLTANKNKPGVKT	CBU_0630	P51752	mip	DRB1*0101, DRB1*0301, DRB1*0401, DRB1*0801, DRB1*1101, DRB1*1301, DRB1*1501
p8	IKGWQEALTRMKPGAIWEI	CBU_0630	P51752	mip	DRB1*0101, DRB1*0301, DRB1*0401, DRB1*0701, DRB1*0801, DRB1*1101, DRB1*1501
p9	AIYFIGWYANLAHIKLGIS	CBU_2065	Q83A45		DRB1*0101, DRB1*0401, DRB1*0701, DRB1*0801, DRB1*1101, DRB1*1301, DRB1*1501
p10	EHTIVVNASASEAAALQ	CBU_1943	Q83AF7	atpA	DRB1*0101, DRB1*0301, DRB1*0401, DRB1*0701, DRB1*0801, DRB1*1301, DRB1*1501
p11	PITKKQLKTMSNYEVIAK	CBU_1869	Q83AL4		DRB1*0101, DRB1*0401, DRB1*0701, DRB1*0801, DRB1*1101, DRB1*1301, DRB1*1501
p12	GKHFDGIKVLKLSPQNTI	CBU_1869	Q83AL4		DRB1*0101, DRB1*0301, DRB1*0401, DRB1*0701, DRB1*0801, DRB1*1101, DRB1*1301, DRB1*1501
p13	FTFRSQDSRRVQEW	CBU_1853	Q83AN0	GtrA family protein	DRB1*0101, DRB1*0301, DRB1*0401, DRB1*0701, DRB1*1101, DRB1*1301, DRB1*1501
p14	PDYVLNAVNHIRYKP	CBU_1835	Q83AP6	protoporphyrinogen oxidase	DRB1*0101, DRB1*0301, DRB1*0401, DRB1*0701, DRB1*0801, DRB1*1101, DRB1*1301, DRB1*1501
p15	MMEHLQNITNLVSTGRQGA	CBU_1835	Q83AP6	protoporphyrinogen oxidase	DRB1*0101, DRB1*0301, DRB1*0401, DRB1*0701, DRB1*0801, DRB1*1101, DRB1*1301, DRB1*1501
p16	LKPFHFISSPTRDLQIA	CBU_1716	Q83B06	gcvT	DRB1*0101, DRB1*0301, DRB1*0401, DRB1*0701, DRB1*0801, DRB1*1101, DRB1*1301, DRB1*1501
p17	KIPVKIIKPPFVRRG	CBU_1716	Q83B06	gcvT	DRB1*0101, DRB1*0401, DRB1*0701, DRB1*1101, DRB1*1301, DRB1*1501
p18	QGHIINIGSISSHQV	CBU_1513	Q83BJ5	short chain dehydrogenase	DRB1*0101, DRB1*0401, DRB1*0701, DRB1*1101, DRB1*1301, DRB1*1501
p19	EAVYKGFTPLKAEDIAEA	CBU_1513	Q83BJ5	short chain dehydrogenase	DRB1*0101, DRB1*0401, DRB1*0701, DRB1*0801, DRB1*1101, DRB1*1301, DRB1*1501
p20	AQPIIHRLSTGQNTNP	CBU_1416	Q83BT6	repressor protein C2	DRB1*0101, DRB1*0401, DRB1*0701, DRB1*0801, DRB1*1101, DRB1*1301, DRB1*1501
p21	IARYFMVNISQLIGEE	CBU_1416	Q83BT6	repressor protein C2	DRB1*0101, DRB1*0301, DRB1*0401, DRB1*0701, DRB1*0801, DRB1*1501
p22	RLGFMSFFTKAVVEALKRF	CBU_1398	Q83BU7	sucB	DRB1*0101, DRB1*0301, DRB1*0401, DRB1*0701, DRB1*0801, DRB1*1101, DRB1*1301, DRB1*1501
p23	REAVLFLVTIKELLEDP	CBU_1398	Q83BU7	sucB	DRB1*0301, DRB1*0401, DRB1*0701, DRB1*0801, DRB1*1301
p24	LPPVTSSVAVKVPSS	CBU_1260	Q83C69	OmpA-like transmembrane domain protein	DRB1*0101, DRB1*0301, DRB1*0401, DRB1*0701, DRB1*1301, DRB1*1501
p25	SDMWQALLAGKSGVK	CBU_0497	Q83E37	fabF	DRB1*0101, DRB1*0401, DRB1*0701, DRB1*0801, DRB1*1101, DRB1*1501
p26	QTQLQQSFSKRTMAT	CBU_1221	Q83CA7	membrane-spanning protein	DRB1*0101, DRB1*0401, DRB1*0701, DRB1*1101, DRB1*1501
p27	RFDLSLMLNYPNSADRY	CBU_1157	Q83CG1		DRB1*0101, DRB1*0301, DRB1*0401, DRB1*0701, DRB1*0801, DRB1*1101, DRB1*1301, DRB1*1501
p28	ISLLVFKNSHRVQLWAK	CBU_1157	Q83CG1		DRB1*0101, DRB1*0301, DRB1*0701, DRB1*0801, DRB1*1101, DRB1*1301, DRB1*1501
p29	VARVSRLKDNFVVLEISKGTEITVQ	CBU_1143	Q83CH2	yajC	DRB1*0101, DRB1*0301, DRB1*0401, DRB1*0701, DRB1*0801, DRB1*1101, DRB1*1501
p30	GTEITVQKASIASVLPK	CBU_1143	Q83CH2	yajC	DRB1*0101, DRB1*0301, DRB1*0401, DRB1*0701, DRB1*1101, DRB1*1301, DRB1*1501
p31	AENVLIIHNKTLAHRYLA	CBU_0968	Q83CY3	phospholipase D	DRB1*0101, DRB1*0301, DRB1*0401, DRB1*0701, DRB1*0801, DRB1*1101, DRB1*1301, DRB1*1501
p32	TGEIVKMINQAKQSIYVQG	CBU_0968	Q83CY3	phospholipase D	DRB1*0101, DRB1*0301, DRB1*0401, DRB1*0801, DRB1*1101, DRB1*1301, DRB1*1501
p33	DGRLEQLNSQNQQLQ	CBU_0891	Q83D52	membrane-associated protein	DRB1*0101, DRB1*0401, DRB1*0701, DRB1*0801, DRB1*1101, DRB1*1501
p34	PAKINLARTYIAMED	CBU_0891	Q83D52	membrane-associated protein	DRB1*0101, DRB1*0301, DRB1*0401, DRB1*0701, DRB1*0801, DRB1*1101, DRB1*1301, DRB1*1501
p35	VFNITLQKVMAPELPVL	CBU_0737	Q83DJ3	tig	DRB1*0101, DRB1*0401, DRB1*0701, DRB1*1101, DRB1*1301, DRB1*1501
p36	IDHLQQMTRQQVAMQTHK	CBU_0737	Q83DJ3	tig	DRB1*0101, DRB1*0301, DRB1*0401, DRB1*0701, DRB1*0801, DRB1*1101, DRB1*1301, DRB1*1501
p37	VAKLRGDLSSIIHKL	CBU_0718	Q83DK8	membrane-associated protein	DRB1*0101, DRB1*0301, DRB1*0401, DRB1*0701, DRB1*1301, DRB1*1501
p38	LSSIIHKLTSFSKTEA	CBU_0718	Q83DK8	membrane-associated protein	DRB1*0101, DRB1*0301, DRB1*0401, DRB1*0701, DRB1*0801, DRB1*1101, DRB1*1301, DRB1*1501
p39	QELFVAQNKAMSDFM	CBU_0612	Q83DT1	ompH	DRB1*0101, DRB1*0301, DRB1*0401, DRB1*0801, DRB1*1101, DRB1*1301, DRB1*1501
p40	QNAFQLQETIVSTEN	CBU_0545	Q83DZ3	lemA	DRB1*0101, DRB1*0301, DRB1*0401, DRB1*0801, DRB1*1101, DRB1*1301, DRB1*1501
p41	LKDVVALRNQAQTAK	CBU_0545	Q83DZ3	lemA	DRB1*0101, DRB1*0301, DRB1*0401, DRB1*0701, DRB1*0801, DRB1*1101, DRB1*1501
p42	DHAYKLAVSSTKSMT	CBU_0497	Q83E37	fabF	DRB1*0101, DRB1*0301, DRB1*0401, DRB1*0701, DRB1*0801, DRB1*1101, DRB1*1301, DRB1*1501
p43	NAGIIRNKLKIQATIN	CBU_0383	Q83EE1	tag	DRB1*0301, DRB1*0801, DRB1*1101, DRB1*1301
p44	GLSWLTILKKRNNYRDSFN	CBU_0383	Q83EE1	tag	DRB1*0301, DRB1*0801, DRB1*1101, DRB1*1301, DRB1*1501
p45	GVAYTYNRANAGLPTNK	CBU_0307	Q83EL2	outer membrane protein	DRB1*0101, DRB1*0301, DRB1*0401, DRB1*0701, DRB1*0801, DRB1*1101, DRB1*1501
p46	VPGYRNASSKRFVAP	CBU_0307	Q83EL2	outer membrane protein	DRB1*0101, DRB1*0301, DRB1*0401, DRB1*0701, DRB1*0801, DRB1*1101, DRB1*1301, DRB1*1501
p47	KAQLIQLKTHVTINAT	CBU_0109	Q83F42	methionine-binding protein	DRB1*0101, DRB1*0301, DRB1*0401, DRB1*0701, DRB1*0801, DRB1*1101, DRB1*1301
p48	SPAVLSAAKKIFGDGA	CBU_0109	Q83F42	methionine-binding protein	DRB1*0301, DRB1*0701, DRB1*0801, DRB1*1101, DRB1*1301, DRB1*1501
p49	DQRITQLKNLNSNNSDSSNDN	CBU_0092	Q83F57	ybgF	DRB1*0101, DRB1*0301, DRB1*0401, DRB1*0701, DRB1*0801, DRB1*1101, DRB1*1301, DRB1*1501
p50	LRPVRYFTGVPSPVKTPE	CBU_1200	Q9ZH99	icd	DRB1*0101, DRB1*0401, DRB1*0701, DRB1*1101, DRB1*1301, DRB1*1501

**Table 3:** HLA class I epitopes selected for immune reactivity screening.

**ID**	**Sequence**	**Source antigen**			**Set^a^**	**Primary HLA I**	**Predicted HLA I restrictions**
		**CBU code**	**UniProt ID**	**Gene name**			
p51	MVEEKYNYFY	CBU_0885	Q83D57		T4SS	A*0101	A*0101, A*0301, A*1101, A*6801, B*3501, B*4403
p52	QSGWTIHHY	CBU_1754	Q83AX3		T4SS	A*0101	A*0101, A*0301, A*1101, B*3501, B*4403
p53	LTLLLNWVNY	CBU_0077	Q83F71	Membrane-spanning protein	T4SS	A*0101	A*0101, A*0301, A*1101, A*6801, B*3501, B*4403
p54	ETYNSINKY	CBU_0794	Q83DE4		T4SS	A*0101	A*0101, A*0301, A*1101, A*6801, B*4403
p55	HTYYLIGFLY	CBU_1457	Q83BP6	Tetratricopeptide repeat family protein	T4SS	A*0101	A*0101, A*0301, A*1101, A*6801, B*4403
p56	YLLDRCPFL	CBU_1754	Q83AX3		T4SS	A*0201	A*0201, A*2402, B*0801
p57	RMYISFFPL	CBU_1543	Q83BG5		T4SS	A*0201	A*0201, A*2402, B*0702, B*0801, B*2705, B*3501
p58	IMIDRLIEQL	CBUA0006	Q45952		T4SS	A*0201	A*0201, A*2402, B*0702, B*0801, B*2705, B*3501
p59	ILDHFYSFL	CBU_1556	Q83BF4	Membrane-spanning protein	T4SS	A*0201	A*0101, A*0201, A*0301, A*2402
p60	LLWHGVSTL	CBU_0077	Q83F71	Membrane-spanning protein	T4SS	A*0201	A*0201, A*2402, B*0702, B*3501
p61	VLDCNYLSK	CBU_0665	Q83DN3		T4SS	A*0301	A*0101, A*0301, A*1101, A*6801
p62	ISYDRFTVGK	CBU_2013	Q83A93		T4SS	A*0301	A*0101, A*0301, A*1101, A*6801
p63	LLSTLCLYRK	CBU_2078	Q83A33	Fic family protein	T4SS	A*0301	A*0101, A*0201, A*0301, A*1101, A*6801
p64	ATSNLIYKLK	CBU_2078	Q83A33	Fic family protein	T4SS	A*0301	A*0101, A*0301, A*1101, A*6801
p65	GVDRFITYK	CBU_0885	Q83D57		T4SS	A*0301	A*0101, A*0301, A*1101, A*6801
p66	QYLQYLWPL	CBU_1457	Q83BP6	Tetratricopeptide repeat family protein	T4SS	A*2402	A*2402, B*0801, B*4403
p67	TYRVLNYKDL	CBU_0665	Q83DN3		T4SS	A*2402	A*2402, B*0702
p68	LYDDEFKFL	CBU_1639	Q83B74		T4SS	A*2402	A*0101, A*2402
p69	SFVPKFFLTF	CBU_2007	Q83A99		T4SS	A*2402	A*2402, B*3501, B*4403
p70	GYHSITPYLF	CBU_0773	Q83DG3	phnB	T4SS	A*2402	A*2402, B*4403
p71	SPHPRVNQF	CBU_1556	Q83BF4	Membrane-spanning protein	T4SS	B*0702	B*0702, *08:02, B*3501, B*4403
p72	FPLGNQFLL	CBU_1314	Q83C21		T4SS	B*0702	A*0201, A*2402, B*0702, B*3501
p73	TPDFPQLFF	CBU_0781	Q83DF6	Ankyrin repeat-containing protein	T4SS	B*0702	A*0101, A*2402, B*0702, B*3501
p74	LPRRHNFYNY	CBUA0006	Q45952		T4SS	B*0702	A*0101, A*0301, B*0702, B*3501
p75	KPLQVINRKY	CBU_2007	Q83A99		T4SS	B*0702	A*0101, B*0702, B*2705, B*3501, B*4403
p76	MEQKLWYPRL	CBU_1677	Q83B41		T4SS	B*4403	A*2402, B*0702, B*4403
p77	FEALHLLYAL	CBU_0175	Q820B6	Serine/threonine protein kinase domain-containing protein	T4SS	B*4403	A*0201, A*2402, B*4403
p78	REMYSRRRMY	CBU_1543	Q83BG5		T4SS	B*4403	A*0101, B*2705, B*4403
p79	IERVFYYFPL	CBU_1079	Q83CN2		T4SS	B*4403	A*2402, B*0702, B*4403
p80	METPPQSPDF	CBU_2052	Q83A58		T4SS	B*4403	A*2402, B*0702, B*3501, B*4403
p81	GSDIIYHNY	CBU_1398	Q83BU7	sucB	S+	A*0101	A*0101, A*0301, A*1101, A*6801
p82	VSDATVAKWY	CBU_1398	Q83BU7	sucB	S+	A*0101	A*0101, A*0301, A*1101, A*6801
p83	KTEQDKLSY	CBU_0630	P51752	mip	S+	A*0101	A*0101, A*0301, A*1101, A*6801
p84	SLESGFTYY	CBU_1260	Q83C69	OmpA-like transmembrane domain protein	S+	A*0101	A*0101, A*0301, A*1101, A*6801, B*4403
p85	FFEDFTHRY	CBU_1835	Q83AP6	Protoporphyrinogen oxidase	S+	A*0101	A*0101, A*0301, A*1101, A*2402, A*6801, B*3501, B*4403
p86	VIDDLIVYRV	CBU_1716	Q83B06	gcvT	S+	A*0201	A*0101, A*0201
p87	FLIAVFARHL	CBU_1967	Q83AD7	Multidrug resistance protein D	S+	A*0201	A*0201, A*2402, B*0702
p88	MLWLPLLLIL	CBU_1143	Q83CH2	yajC	S+	A*0201	A*0201, A*2402, B*3501
p89	FLFFYSGLIL	CBU_1967	Q83AD7	Multidrug resistance protein D	S+	A*0201	A*0201, A*0301, A*2402
p90	NMFQHLPYL	CBU_0109	Q83F42	Methionine-binding protein	S+	A*0201	A*0201, A*2402, B*2705, B*3501
p91	KTFVYPMGLY	CBU_0109	Q83F42	Methionine-binding protein	S+	A*0301	A*0101, A*0301, A*1101, A*6801, B*2705, B*4403
p92	ASFQNYLNDY	CBU_0092	Q83F57	ybgF	S+	A*0301	A*0101, A*0301, A*1101, A*6801, B*2705, B*4403
p93	KLILFVYRY	CBU_1853	Q83AN0	GtrA family protein	S+	A*0301	A*0101, A*0301, A*1101, A*6801, B*2705, B*3501, B*4403
p94	SLFANMNGHY	CBU_0754	Q83DH7	Acriflavin resistance protein	S+	A*0301	A*0101, A*0301, A*1101, A*6801, B*4403
p95	LSATFITSK	CBU_1835	Q83AP6	Protoporphyrinogen oxidase	S+	A*0301	A*0101, A*0301, A*1101, A*6801
p96	WYVVNNNYL	CBU_1943	Q83AF7	atpA	S+	A*2402	A*2402, B*3501, B*4403
p97	NFSDYIWHF	CBU_0383	Q83EE1	tag	S+	A*2402	A*0101, A*2402, B*4403
p98	IYDDRLLFEF	CBU_0383	Q83EE1	tag	S+	A*2402	A*0101, A*2402
p99	SFNGWIAYL	CBU_0891	Q83D52	Membrane-associated protein	S+	A*2402	A*2402, B*2705, B*4403
p100	TYRHNKKLL	CBU_2065	Q83A45		S+	A*2402	A*2402, B*0702
p101	LPIDNFYAF	CBU_1260	Q83C69	OmpA-like transmembrane domain protein	S+	B*0702	A*2402, B*0702, B*0801, B*3501
p102	LPRNRYRLVF	CBU_1869	Q83AL4		S+	B*0702	A*2402, B*0702, B*0801, B*3501
p103	LPQKPWWKF	CBU_2065	Q83A45		S+	B*0702	A*2402, B*0702, B*3501
p104	APVTHLFTV	CBU_0307	Q83EL2	Outer membrane protein	S+	B*0702	A*0201, B*0702, B*3501
p105	LPNHFAPQL	CBU_0754	Q83DH7	Acriflavin resistance protein	S+	B*0702	A*2402, B*0702, B*3501
p106	EEFVKTQSY	CBU_1943	Q83AF7	atpA	S+	B*4403	A*0101, B*4403
p107	MEVRDLLNSY	CBU_0236	Q83ES6	tufA	S+	B*4403	A*0101, B*4403
p108	GEEATAFLRY	CBU_1716	Q83B06	gcvT	S+	B*4403	A*0101, B*4403
p109	IEVVKKYMDY	CBU_0545	Q83DZ3	lemA	S+	B*4403	A*0101, B*4403
p110	NEIETATRY	CBU_0664	Q81ZP4	Transposase	S+	B*4403	A*0101, B*4403
p111	VTLVEFFDY	CBU_1910	H7C7D7	com1	S+	A*0101	A*0101, A*0301, A*1101, A*6801, B*3501
p112	YYAFHDALL	CBU_1910	H7C7D7	com1	S+	A*2402	A*2402
p113	KDIQSIVHHY	CBU_1910	H7C7D7	com1	S+	B*4403	A*0101, B*2705, B*4403
p114	TPTFVIGNKA	CBU_1910	H7C7D7	com1	S+	B*0702	B*0702, B*3501
p115	YLVNHPEVL	CBU_1910	H7C7D7	com1	S+	A*0201	A*0201, A*2402

a *Antigen set: substrates of the type IV secretion system (T4SS). Sero-reactive antigens (S+) based on antibody recognition in immune human sera*.

### HLA Binding of Predicted *C. burnetii* Epitopes

**Figure 1:**
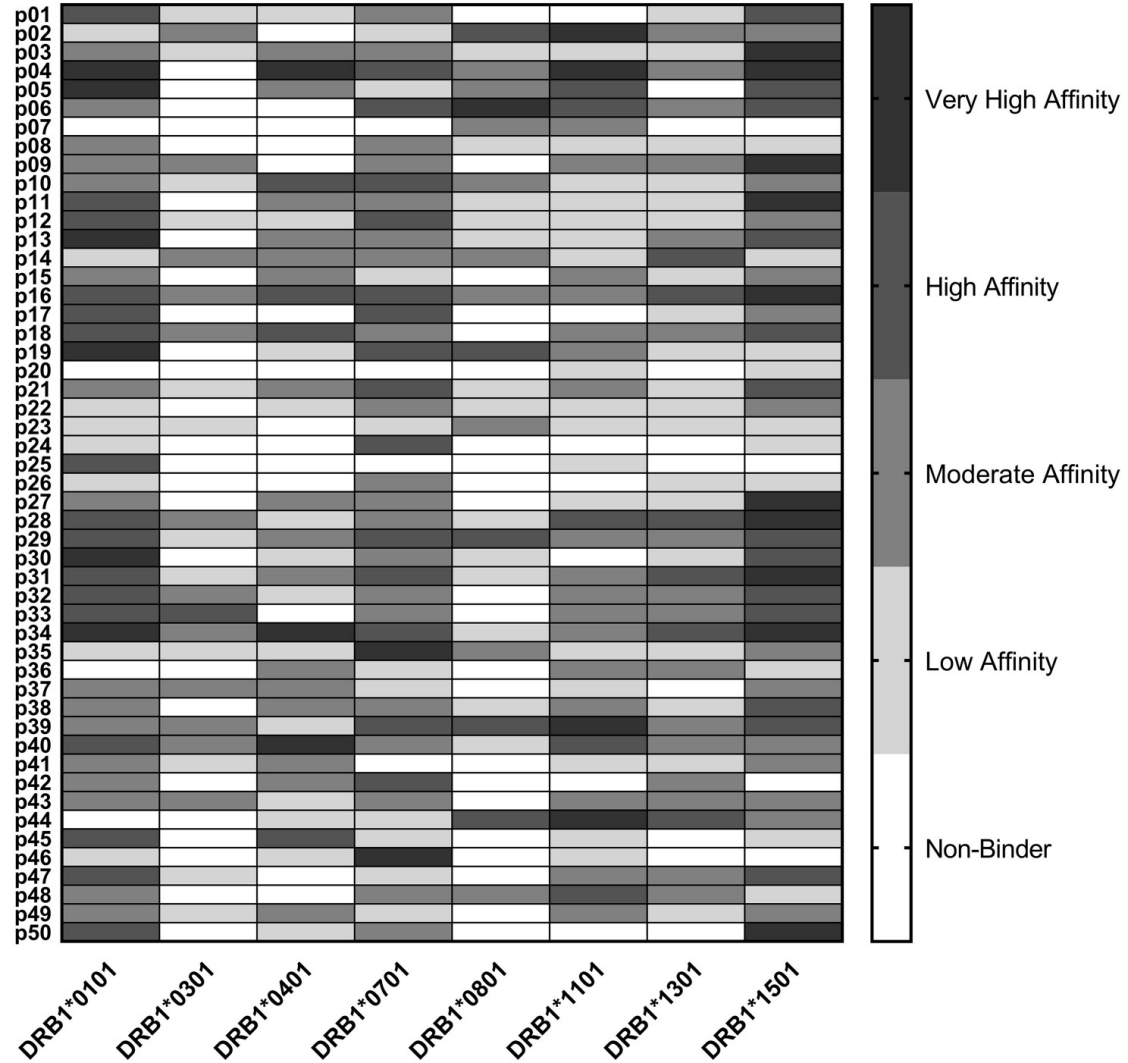
HLA Class II Binding Assay Results. *C. burnetii* HLA class II epitope affinities for eight common DR alleles were assessed in competition binding assays. Peptide binding strength was classified according to IC_50_ value in the following affinity categories: very high (<0.1 μM), high (0.1–1 μM), moderate (1–10 μM), and low (10–100 μM). Epitopes with IC_50_ values >100 μM or with no dose-dependent response were considered non-binders.

Epitope predictions were validated using HLA binding assays. Peptides representing the 50 promiscuous *C. burnetii* HLA class II epitope clusters were assayed in competition binding assays against each of the eight class II HLA supertype alleles (Figure 1). Each of the peptides bound as predicted to at least two HLA-DR alleles: 6% bound to two HLA-DR alleles, 2% to three alleles, 10% to four alleles, 4% to five alleles, 28% to six alleles, 24% to seven alleles, and 26% to all eight alleles. Independent of original binding predictions, amongst all 400 peptide-allele pairs tested, 6.5% of peptides bound with very high affinity (IC_50_ < 0.1 μM), 16% of peptides bound with high affinity (IC_50_ 0.1–1 μM), 30% bound with moderate affinity (IC_50_ 1–10 μM), 25.5% bound with low affinity (IC_50_ 10–100 μM), and 22% exhibited no detectable affinity for the HLA-DR tested (IC_50_ >100 μM or no dose-dependent response). Overall, 81% of predicted binding events and 41% of predicted non-binding events were verified *in vitro*. Collectively, the agreement of computational predictions with binding assay results was 75% (χ^2^, *p* < 0.001), ranging from 60 to 88% for individual alleles (Table S1), consistent with published studies using the same algorithms and assay conditions (57, 58).

**Figure 2:**
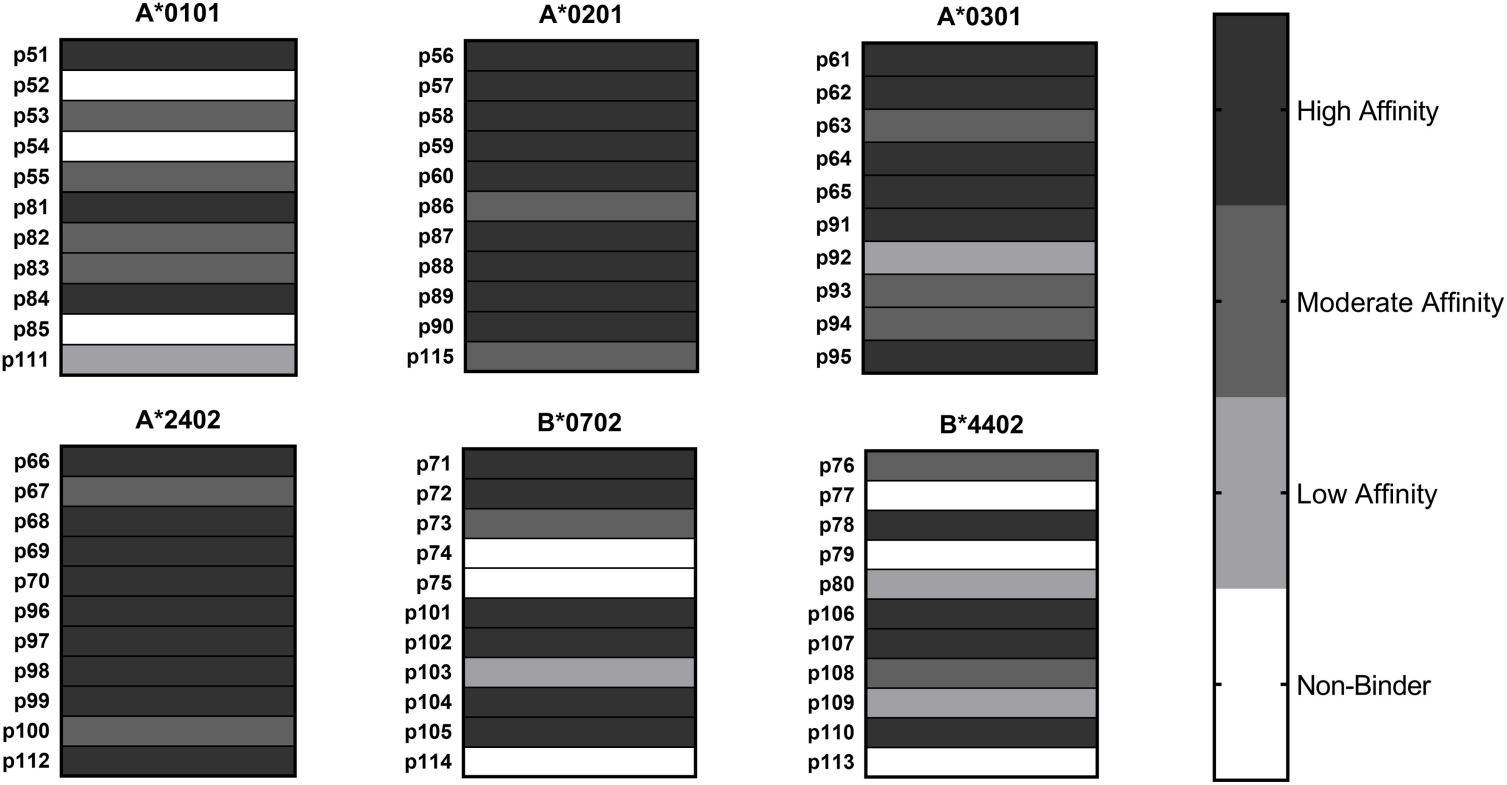
HLA Class I Binding Assay Results. *C. burnetii* HLA class I epitope affinities for six supertype HLA-A and HLA-B alleles were assessed in competition binding assays. Peptide binding strength was classified according to IC_50_ value in the following categories: high affinity (<5 μM), moderate affinity (5–50 μM), and low affinity (50–1,000 μM). Epitopes with IC_50_ values >1,000 μM or with no dose-dependent response were considered non-binders.

HLA class I binding affinities were determined for the primary predicted HLA-A/B supertype allele for each peptide (Figure 2). Among the 65 peptide-allele pairs tested, 56% bound with high affinity (IC_50_ < 5 μM), 21.5% with moderate affinity (IC_50_ 5–50 μM), 7.5% with low affinity (IC_50_ 50–1,000 μM), and 14% exhibited no detectable affinity for the HLA class I allele tested (IC_50_ >1,000 μM or no dose-dependent response). All predicted A^*^0201, A^*^0301, and A^*^2402 peptides, and 8/11 A^*^0101 and B^*^0702 peptides bound HLA as predicted. An assay using B^*^4403 molecules was not available, but an assay using the related B^*^4402 allele showed eight out of 11 B^*^4403 peptides bound HLA as predicted. Based on these results, epitope prediction accuracy was 100% for A^*^0201, A^*^0301, and A^*^2402 and 73% for A^*^0101, B^*^0702, and B^*^4403. Overall, predictive accuracy for this set of HLA class I peptides was 89% (Table S1). Taken together, the binding assay results suggest that the predicted HLA class II epitope clusters and HLA class I epitopes represent a set of sequences with meaningful potential for stimulating *C. burnetii*-specific immune responses across a broad range of HLA types.

### Immunogenicity of Class II Epitopes in tgHLA-DR3 Mice

While peptide binding to HLA is necessary to induce a T-cell response, it is not sufficient. To determine whether the predicted *C. burnetii* HLA class II epitope clusters are capable of eliciting a *de novo* immune response via a cognate human HLA *in vivo*, the class II peptides were screened for immunogenicity in transgenic mice expressing human HLA-DR3 (tgHLA-DR3). The 50 HLA class II *C. burnetii* peptides were arranged into five groups of 10 peptides each based on predicted HLA-DR3 immunogenicity and three mice per group were subjected to heterologous DNA/DNA/peptide/peptide prime-boost immunizations. Peptide-specific responses of splenocytes from tgHLA-DR3 mice were assessed by *ex vivo* IFNγ ELISpot assay. Positive peptide-specific IFNγ responses *in vivo* were observed for 11 peptides, eight of which evoked responses in at least 2/3 mice (Table 4). All 11 of these immunogenic peptides were predicted to bind HLA-DR3, and all except one (p36) also showed binding to DRB1^*^0301 *in vitro*. Therefore, altogether 10/21 peptides both predicted and confirmed to bind HLA-DR3 were immunogenic *in vivo*. None of the 13 peptides that were predicted not to bind HLA-DR3 were immunogenic. An odds ratio, calculated to determine the association of epitope prediction and immunogenicity, showed a statistically significant association for immunogenicity given an HLA-DR3 prediction (Fisher's exact test, *p* = 0.0229). No peptide-specific responses were detected in mock-immunized mice that received vehicle vaccine controls.

**Table 4:** *C. burnetii* HLA class II epitope immunogenicity in tgHLA-DR3 mice.

**Peptide ID**	**No. of mice responding^**d**^**	**Mouse 1/3**			**Mouse 2/3**			**Mouse 3/3**		
		**SFU^**a**^**	**SI^**b**^**	***p*-value^**c**^**	**SFU^**a**^**	**SI^**b**^**	***p*-value^**c**^**	**SFU^**a**^**	**SI^**b**^**	***p*-value^**c**^**
p2	2	120	6.63	0.0003	141	8.28	< 0.0001	18	1.53	0.1384
p10	2	256	5.80	< 0.0001	82	4.51	0.0043	43	2.00	0.0496
p14	2	157	7.94	0.0006	371	20.21	0.0001	11	1.42	0.0149
p16	1	180	8.94	0.0007	27	2.05	0.0060	19	1.94	0.0164
p23	2	69	2.30	0.0038	21	1.89	0.0372	63	2.47	0.0172
p28	2	63	3.76	0.0013	54	3.79	0.0094	41	2.63	0.0227
p31	1	0	0.88	0.1143	14	1.72	0.0621	84	4.32	0.0007
p32	2	64	3.82	0.0053	70	4.62	0.0103	28	2.11	0.0609
p36	2	52	3.44	0.0468	55	3.86	0.0193	0	0.86	0.2215
p41	1	87	5.06	0.0052	30	2.55	0.0433	0	0.98	0.3566
p43	3	135	4.88	0.0001	65	3.04	0.0013	116	4.48	0.0001

a Average number of spot forming units (SFU) per million splenocytes from triplicate wells; the average number of SFU/million in medium only control wells was subtracted. Negative values were assigned 0.

b Stimulation index (SI) calculated as average number of SFUs in peptide stimulated wells over medium only control wells.

c SFUs in peptide stimulated wells from immunized mice compared to mock-immunized mice by Student's t-test.

d *Mice are deemed responsive to a peptide if their SFU is >50 and if their SI is >2 and if their p-value is lower than 0.05*.

### Selection of *Coxiella*-Exposed Human Subjects for Epitope Antigenicity Screening

The transgenic mouse study provided a snapshot of peptide-specific immunogenicity for a single HLA class II allele. To assess antigenicity across multiple HLA types and determine whether the selected class I and II peptides are capable of recalling long-lasting IFNγ memory responses, we next turned to a unique cohort of individuals naturally exposed to *C. burnetii*. Subjects for this study were recruited from a well-characterized population in the village of Herpen in the Netherlands (48), which experienced a high incidence of *C. burnetii* infection during the 2007–2010 Q fever outbreak. Recruited subjects were categorized based on clinical Q fever history and recall responses to heat-killed *C. burnetii* in a whole blood IFNγ release assay (IGRA) (Table 5). Group A and B subjects had no clinical history of Q fever, while group C individuals had recovered from an acute episode of diagnosed Q fever. Group A controls had no IFNγ recall responses to *C. burnetii*. Group B (past asymptomatic infection) and group C (past symptomatic infection) subjects showed varying degrees of IFNγ recall responses to *C. burnetii* (both *p* < 0.0001 compared to group A controls by one-way ANOVA with Holm-Šídák multiple comparisons test), with no significant difference between the two groups (*p* = 0.9). HLA types were sufficiently diverse within the total cohort to have all desired HLA types represented in all three clinical groups (Tables S2, S3), with the exception of HLA-DR8, which is underrepresented particularly in group C, in accordance with this allele's expected low frequency in the Dutch population (42, 59, 60). Assessment of the binding potential of all peptides with respect to the HLA types of the 136 subjects in groups A-C showed no significant differences across groups, with 75–82% of all class I peptides and between 93 and 96% of all class II peptides predicted to be recognized by each group.

**Table 5:** Categorization of human study subjects.

**Group**	***N***	**Age in years (median, IQR)^**a**^**	**Females *N* (%)**	**Coxiella-specific IFNγ response in pg/ml (median, IQR)^**b**^**	**Previous clinical Q fever episode^**c**^**
A (controls)	26	55 [47-57]	14 (54%)	3 [1–10.3]	–
B (past asymptomatic)	73	54 [47-63]	47 (64%)	330 [168–660]	–
C (past symptomatic)	37	54 [48-63]	20 (54%)	348 [180–717]	+

a At inclusion into the study in October 2015.

b At inclusion into the study in October 2015, medium only background subtracted.

c *Either formally notified or self-reported (48)*.

Two partially overlapping subgroups of the total cohort were selected based on a broad representation of HLA-A/HLA-B and HLA-DR supertypes and allocated to HLA class I and II peptide screening, respectively. The screening cohort for the 50 HLA class II peptides consisted of 21 group A control donors (IGRA-, no clinical disease), and 56 IGRA+ donors from groups B (asymptomatic, *n* = 33) and C (symptomatic, *n* = 23) (Table S2). The screening cohort for the 65 HLA class I peptides included 20 group A control donors (IGRA-, no clinical disease), and 57 IGRA+ donors from groups B (asymptomatic, *n* = 32) and C (symptomatic, *n* = 25) (Table S3). Care was taken to maintain a distribution of IGRA responses comparable to the total cohort (Tables S2, S3).

### Human Antigenic Responses to Predicted HLA Class II Epitopes

Human antigenic T-cell IFNγ recall responses against the predicted epitope clusters from *C. burnetii* were evaluated *ex vivo* using freshly isolated peripheral blood mononuclear cells (PBMCs) in a cultured ELISpot assay for detection of central memory T-cells. A cultured ELISpot assay was used to achieve high sensitivity for detecting low frequency antigen-specific T-cell responses. Amongst the 3,843 assessments made for the HLA class II peptides (50 peptides screened against 77 donors with some exceptions due to technical error or insufficient cell numbers), 307 yielded positive responses [stimulation index (SI) ≥2, Figures S1, S2]. These antigenic responses covered 44/50 HLA class II peptides. Only six peptides were not recognized by any subject (p9, p11, p34, p35, p40, and p49). For four of these peptides, a second peptide from the same respective source antigen induced recall responses by at least one IGRA+ subject. Therefore, responses were detected to all but two (CBU_2065 and CBU_0092/YbgF) of the 29 source antigens from which HLA class II epitope clusters were predicted and screened.

**Figure 3:**
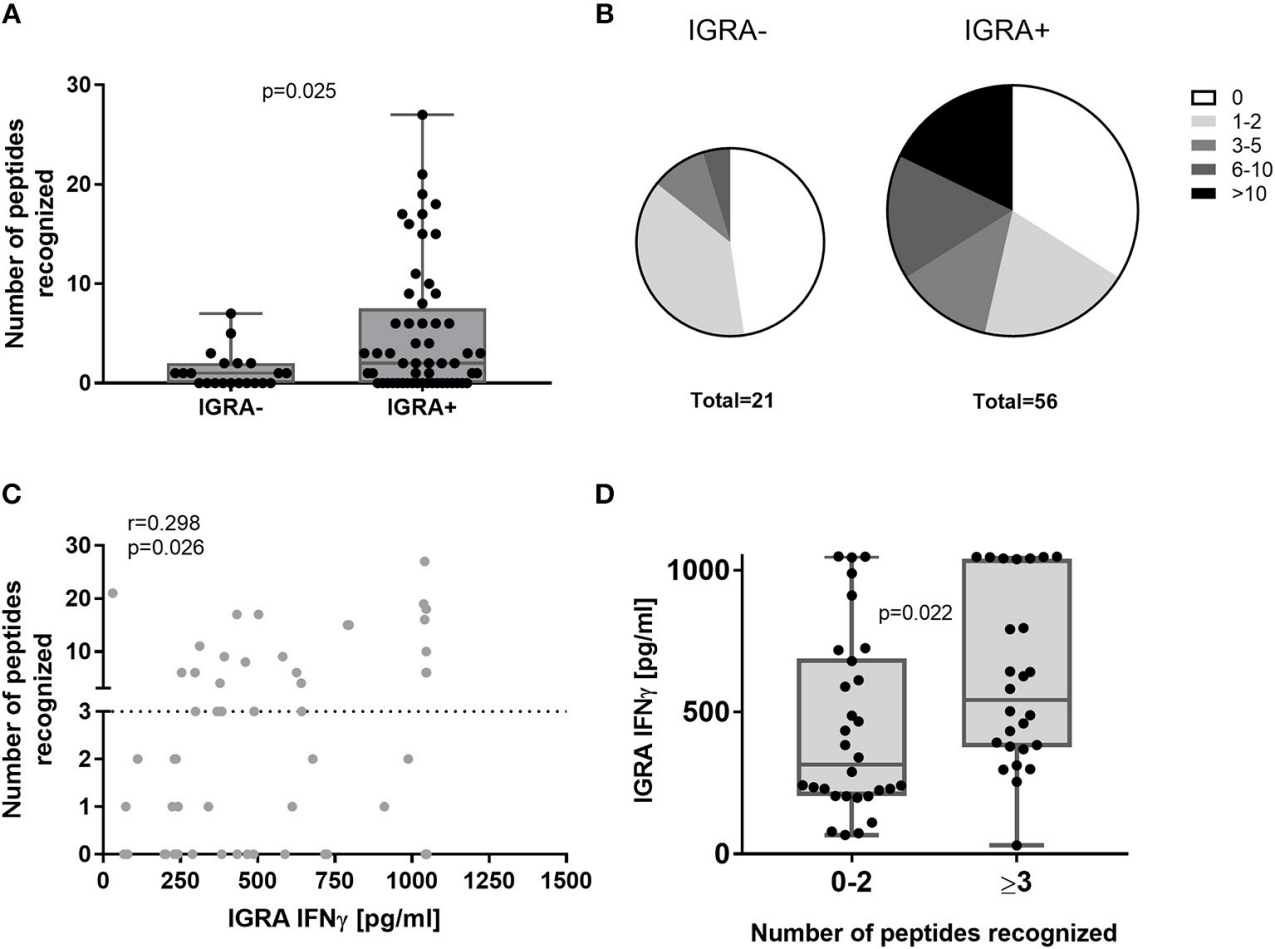
Cumulative HLA class II peptide responses across clinical groups and in relation to IGRA. Data are shown for IGRA- and IGRA+ as the cumulative peptide response (SI ≥2) per donor **(A)** or as the proportion of subjects recognizing 0, 1–2, 3–5, 6–10, or >10 peptides **(B)**. **(C)** The cumulative number of peptides recognized by individual IGRA+ subjects is plotted against their IGRA response to whole heat-killed *C. burnetii* and analyzed by Spearman correlation. **(D)** IGRA responses are shown for IGRA+ individuals recognizing 0–2 or ≥3 peptides (cut-off SI≥2). Groups in **(A,D)** were compared by Mann-Whitney-test. Whisker-dot-plots show the median and interquartile range (25 and 75th percentile) with whiskers extending from min to max values.

The cumulative HLA class II peptide response per subject was more frequent in IGRA+ compared to IGRA- subjects (Figure 3A). The majority of IGRA- individuals recognized none (10/21 donors) or only one or two peptides (8/21 donors). While only 14% (3/21) of IGRA- group A individuals recognized three or more peptides, this was true for 46% (26/56) IGRA+ group B and C donors (Figure 3B). Amongst IGRA+ individuals, there was a positive correlation between the cumulative number of recognized peptides per donor, and the IFNγ responses to whole heat-killed *C. burnetii* as assessed 12–17 months prior to peptide screening (Figure 3C). Of note, all but one individual that showed responses to three or more peptides had an IGRA response of at least 250 pg/ml IFNγ in this standardized assay (Figure 3D).

**Figure 4:**
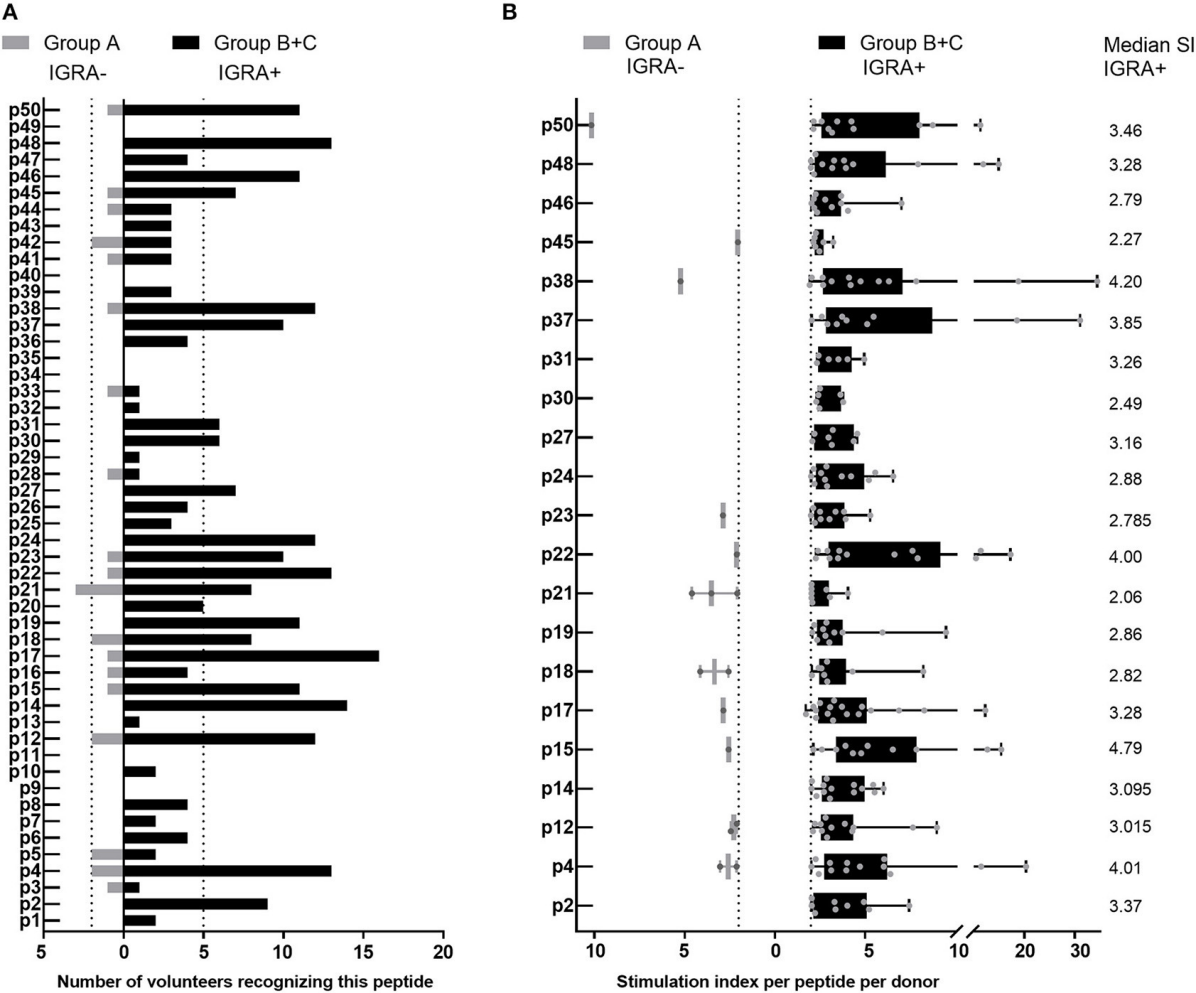
Highly antigenic HLA class II peptides identified by cultured IFNγ ELISpot assay. Data are shown as the number of individuals recognizing the 50 individual peptides, depending on whether subjects are IGRA- (Group A, no clinical disease, *n* = 21; gray bars) or IGRA+ (Group B, asymptomatic; Group C, symptomatic; *n* = 56; black bars). **(A)** Shows peptide responses reaching a SI ≥2. Bars extending over dotted lines indicate those peptides that were recognized by more than 10% of IGRA- subjects (>2/21) or IGRA+ subjects (>5/56). **(B)** Shows the stimulation index for each positive response per subject for the 21 highly antigenic peptides identified in **(A)**. Whisker-dot-plots show the interquartile range (25 and 75th percentile) with whiskers extending from min to max values. Numbers indicate the median SI per peptide for IGRA+ subjects.

Of the 44 antigenic HLA class II peptides, 21 peptides were recognized by >10% of all IGRA+ individuals (at least 6/56, Figure 4A) and were hence considered highly antigenic. These 21 highly antigenic peptides accounted for 78.3% of all positive peptide responses of IGRA+ individuals that reached a SI≥2 (220/281). A large proportion of these responses was well above the cut-off of SI ≥2: Amongst responding IGRA+ individuals, responses to 13 of these 21 peptides showed a median SI between 3.0 and 4.8 (Figure 4B), and for 11 of these peptides >10% of all IGRA+ donors reached at least SI ≥3. Amongst IGRA- control subjects group A, these peptides elicited either no recall responses (11/21), or responses from only one or two individuals. Only for a single peptide (p21) did the recall response frequency reach a similar proportion in IGRA- (3/21; 14.3%) as in IGRA+ donors (8/56; 14.3%). Amongst the 21 highly antigenic peptides, there were five source proteins that were represented by two epitopes each (p14+p15, CBU_1835/protoporphyrinogen oxidase; p18+p19, CBU_1513/short chain dehydrogenase; p22+p23, CBU_1398/SucB, p37+p38, CBU_0718, p45+p46, CBU_0307/outer membrane protein). Notably, not only were all 11 of the tgHLA-DR3 mice immunogenic peptides also antigenic in at least one IGRA+ individual, but five of the tgHLA-DR3 immunogenic peptides were amongst the 21 highly reactive peptides in the human cohort (p2, p14, p16, p22, p31).

**Figure 5:**
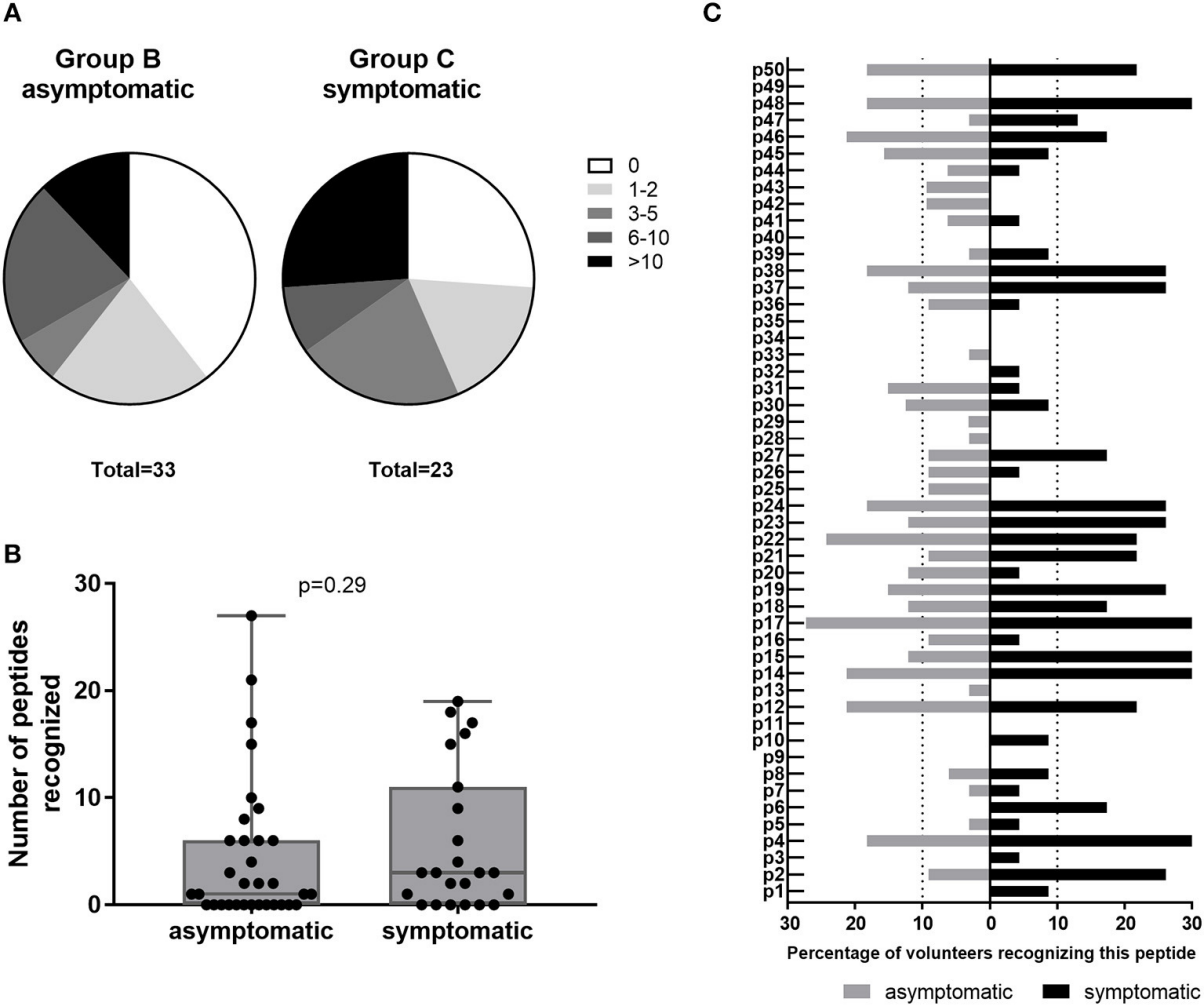
Class II peptide antigenicity patterns in past asymptomatic and symptomatic, IGRA+ individuals. Data are shown as **(A)** the proportion of subjects recognizing 0, 1–2, 3–5, 6–10, or >10 peptides for IGRA+ donors with past asymptomatic or symptomatic Q fever or **(B)** the cumulative peptide response (SI ≥2) per donor. Groups are compared by Mann-Whitney-test. Whisker-dot-plots show the median, 25 and 75th percentile with whiskers extending from min to max values. **(C)** Data are shown as the proportion of subjects recognizing the 50 individual peptides reaching a SI ≥2, depending on whether IGRA+ individuals were asymptomatic (Group B, *n* = 32; gray bars) or symptomatic (Group C, *n* = 23; black bars) during the Q fever outbreak. Bars extending over dotted lines indicate those peptides that were recognized by more than 10% of group B (>3/32) or group C subjects (>2/23).

Amongst IGRA+ donors, group C of past symptomatic individuals had a higher proportion of high responders (recognizing >10 peptides) and a smaller proportion of non-responders to HLA class II epitope clusters than past asymptomatic subjects (Group B, Figure 5A). However, the two groups did not differ significantly in their cumulative peptide response per subject (Figure 5B). When comparing responses to individual peptides between group B and C, there was again a trend that responses particularly to the most antigenic peptides were more frequent amongst past symptomatic donors and a number of peptides were recognized only within this group by either a single (p3, p33) or multiple donors (p1, p6, p10) (Figure 5C). There were some peptides, however, that were recognized solely by past asymptomatic subjects (p25, p42, and p43, 3/33 each; p13, p28, p29, and p33, 1/33 each).

### Human Antigenic Responses to Predicted HLA Class I Epitopes

Recall responses to the 65 HLA class I peptides were also assessed using the cultured IFNγ ELISpot approach. In 4,794 assessments (65 peptides screened in 77 donors with some exceptions due to technical error or insufficient cell numbers), only 23 positive responses were detected (Figure S3). Four of these positive responses came from IGRA- group A control subjects. The remaining 19 responses were detected in IGRA+ subjects, with one individual recognizing 8 different peptides (Figures S3, S4). Of the 15 HLA class I peptides that were found to be antigenic in humans, ten peptides were recognized by only a single donor each and only five HLA class I peptides were recognized more than once: p53, p91, and p92 were each recognized by 2/57 donors, and two epitopes (p111 and p113) from com1 were recognized by 3/57 and 4/57 donors, respectively. Only two of the reactive peptides came from the T4SS data set (p53, p54), the remaining 13 were derived from sero-reactive source antigens. Albeit rare, HLA class I peptide responses were clearly detectable, with nearly half (11/23) of the positive responses reaching a stimulation index (SI) of ≥3 (Figures S3, S4), and all responses were consistent with predictions for primary or secondary HLA-A/B allele binding.

### Evaluation of Class I and Class II Epitopes in Guinea Pig Model of Vaccine Reactogenicity

Due to reactogenicity issues observed with the only Q fever vaccine licensed for humans, all *C. burnetii* HLA class I and class II peptides examined in this study were screened for potential reactogenicity in a guinea pig model of exposure-primed delayed-type hypersensitivity (55, 61), with the goal of eliminating reactogenic peptides from further consideration for inclusion in a vaccine. In contrast to reactogenicity mediated by the whole cell phase I vaccine COXEVAC®, no gross reactions were noted at any of the negative control or peptide pool injection sites (Figure S5). Mild lymphocytic inflammation (score = 1) was noted in two HLA class II peptide pools; no overlap in peptides was noted between the two pools and no other pools with the same peptides showed histological changes. Therefore, these reactions were not likely to be considered a reactogenic response to *C. burnetii* peptides.

## Discussion

In the present study, we aimed to identify HLA class I and class II epitopes for inclusion in a non-reactogenic T-cell-targeted vaccine to control *C. burnetii* infection and hence prevent Q fever disease in humans. HLA binding competition assays demonstrated a high accuracy of the immunoinformatic predictions for HLA class I and class II recognition, and a murine immunogenicity study demonstrated that half of the peptides both predicted and shown to bind HLA-DR3 were also capable of eliciting *de novo* responses in tgHLA-DR3 mice. Notably, while responses to the 65 selected HLA class I epitopes were barely detectable in PBMCs from individuals naturally exposed to *C. burnetii* during the Dutch Q fever outbreak 7–10 years prior to this study, 21 out of the 50 predicted HLA class II epitope clusters recalled responses in 10–28% of those individuals that also showed an *ex vivo* response to whole heat-killed *C. burnetii*. We thereby demonstrate for the first time that natural exposure of humans to *C. burnetii* induces long-lived responses to immunodominant, promiscuous, and conserved HLA class II T-cell epitope clusters. These peptide sequences are desirable candidates for inclusion in a vaccine aiming to elicit sustained T-cell memory in a broad and immunogenetically diverse human population that can be boosted and recalled by natural exposure.

These long-lived epitope-specific T-cell responses are consistent with previous studies in Q fever convalescent individuals and recipients of the whole cell formalin-inactivated phase I vaccine Q-VAX®, which have consistently reported a long-lived T-cell response to whole cell *C. burnetii* antigen. The T-cell response, in this context, develops within 2 weeks post-vaccination (62), is detectable for at least 8–10 years following natural exposure or vaccination (63, 64) and in some studies was found to be considerably more durable than the mounted antibody response (62, 63). However, studies investigating the specific antigen targets of the induced immune response in humans have so far solely focused on antibodies (20, 21, 23, 24, 26, 27, 29, 33, 65–71). T-cell antigen targets have only been assessed in a limited number of murine studies (18, 21, 22, 72), including one study that investigated T-cell responses to recombinant *C. burnetii* proteins by ELISpot in HLA-DR4 transgenic mice (21). T-cell antigen targets of murine CD4 T-cells in these studies were all chosen based on antigen recognition by human sera of Q fever exposed individuals (18, 21, 22). While this B-cell antigen-guided screening approach for T-cell targets precludes identification of T-cell epitopes for source antigens that do not elicit a dominant antibody response, it has the advantage of significantly reducing and focusing the number of antigens to be evaluated. In the present study, 44/50 HLA class II peptides (88%) from 27/29 selected sero-reactive source antigens (93%) elicited T-cell recall responses from at least one donor with a history of *C. burnetii* exposure during the Dutch Q fever outbreak. This large hit rate for HLA class II epitope clusters illustrated the feasibility of this approach to focus on sero-reactive antigens for T-cell epitope prediction.

One major challenge when focusing a vaccine on individual epitopes is to choose epitopes that are capable of eliciting responses from a large part of the human population. Despite the expected HLA diversity in this human cohort, 21/50 (42%) of the screened HLA class II peptides, representing 16/29 source antigens, were recognized by more than 10% of the subjects exposed 7–10 years prior. In comparison, IFNγ recall responses to H2 I-Ab-restricted *C. burnetii* epitopes were only detectable for 17–26 and 5.3–6.8% of the predicted epitopes in inbred C57BL/6 mice immediately following immunization with the selected source antigens or whole cell antigen, respectively (18, 22). Of note, all but one of the antigens in the murine CD4 epitope vaccine study (18) were also amongst the HLA class II source antigens in the present study, and one of the seven vaccine epitope sequences (OmpA146–160), covered by p45 in our peptide screening library, was one of the highly antigenic peptides recognized by 7/56 (12.5%) of IGRA+ subjects. These results validate the ability of the immunoinformatic algorithms employed to predict promiscuous HLA class II epitopes for humans. Moreover, the highly antigenic HLA class II epitopes identified herein are not only promiscuous across HLA-DR supertypes (76% predicted binding to 6 or more alleles) but are also conserved across all *C. burnetii* genomes publicly available in 2015, as well as in the Dutch outbreak strain (39). Such conserved immunodominant T-cell epitopes have also been found for *Mycobacterium tuberculosis* (73–75), hepatitis C virus (76, 77), and HIV (78, 79) and are interesting vaccine targets, since they are likely under positive selection pressure and hence unlikely to be subject to vaccine-induced immune evasion (73).

A somewhat unexpected finding of the present study was the scarcity of detectable responses to the predicted HLA class I epitopes in human subjects. Earlier studies indicated that human CD8 T-cells contribute to *C. burnetii*-specific IFNγ production (80), and in murine models CD8 T-cells appear to be more critical than CD4 T-cells for resolving infection (15, 81). Using a similar approach to the present study, a recent report identified 29 class I epitopes from T4SS substrates that conferred partial protection in a murine vaccine-challenge study (72). One of these 29 was amongst the few HLA class I epitopes recognized by two human donors (p92 from the tol-pal system protein YbgF). The proportion of peptides recognized at least once in the human cohort (15/65, 23%) was comparable to the recognition frequency observed in the murine study (29/157, 18%), suggesting that human HLA class I epitopes were predicted effectively in the present study.

The much smaller frequency of HLA class I compared to HLA class II responses could be due to a number of reasons. Firstly, while HLA class II peptides are promiscuous epitope clusters each containing 5–20 9-mers with various HLA-DR binding motifs, HLA class I epitopes do not display this promiscuity. Each of the 65 HLA class I peptides have, on average, 0.4 binding motifs across the class I supertype alleles modeled by EpiMatrix and represented in over 95% of the global human population. This is in stark contrast to 1.36 binding motifs found, on average, for the 50 HLA class II peptides across class II supertype alleles, representing a 3.4-fold increase over the number of motifs for the HLA class I peptides. The larger number of binding opportunities for HLA class II peptides may thus contribute to the greater number of responses they recalled.

Secondly, reports have shown that the TCR repertoire of CD4 T-cells is estimated to be about five times greater than the TCR repertoire of CD8 T-cells (82), suggesting that there are more opportunities for CD4 T-cell reactivity. Finally, it is also possible that circulating *C. burnetii*-specific CD8 T-cells are less well detected than CD4 T-cells at such a late time point (7–10 years) post-exposure, which would not be unprecedented: following smallpox infection or vaccination, antigen-specific CD8 T-cells show a much faster contraction to undetectable levels than do CD4 T-cells (83, 84). Similarly, a significant contraction in *Mycobacterium tuberculosis*-specific CD8 but not CD4 effector memory responses was reported (85) as well as a significant decrease in total and antigen-specific central memory CD8 T-cells (86). Of note, *C. burnetii* epitope-specific murine CD8 T-cell responses were assessed only 10 days post-infection (72), and challenge experiments were carried out no later than 28 days following vaccination or transfer of infection-induced CD8 T-cells (15, 72). The most obvious starting point for evaluating whether responses to a common set of human HLA class I epitopes are induced by natural exposure to *C. burnetii* would therefore be to screen in much more recently exposed donors. In the absence of another outbreak, inclusion of a sufficiently large cohort in a reasonable time frame is, however, unlikely. Alternatively, one could assess HLA class I epitope responses in individuals that recently received the whole cell vaccine Q-VAX®. This would provide additional insights into the contribution of the predicted epitopes to protection, which would also be valuable for the identified highly reactive HLA class II epitope clusters. Given that the vaccine is only licensed for use in Australia, however, pursuit of such a study would be restricted to this location. Finally, we cannot strictly conclude that the observed HLA class II recall responses arise solely from CD4 T-cells and not CD8 T-cells. Although rare, HLA class II-restricted CD8 T-cells have been identified in both mice (87) and humans (88). Definitively addressing the relative responses would require further experimentation with purified T-cell populations rather than total PBMCs.

In the present study, we further compared epitope-specific responses between *C. burnetii* exposed individuals with or without a history of an acute clinical episode of Q fever. Although responses tended to be more frequent in past symptomatic donors and some peptides were recognized only by either past symptomatic or asymptomatic individuals, these observations are based on a relatively small number of responding subjects. Moreover, none of the observed differences were striking enough to suggest a significant difference in the quality of the induced T-cell response between these two groups that would explain why some individuals developed symptoms and others did not. What would be of interest in future studies is to compare this group of convalescent individuals with patients that developed persistent infection with *C. burnetii* (chronic Q fever) (1, 89), to identify those epitopes for which recognition may be crucial to control and clear infection and hence might be lacking in individuals with persistent infection. This would be analogous to observations from herpes simplex virus vaccine research, where distinct epitope-specific T-cell repertoires have been identified for infected individuals that either successfully control infection (asymptomatic) or not, leading to a proposed emphasis on so-called “asymptomatic epitopes” (90).

The ultimate aim of the Q-VaxCelerate consortium is to develop a non-reactogenic Q fever vaccine that could be administered without screening for pre-exposure, especially in an outbreak setting or when occupational hazard warrants immediate broad-scale vaccination. Since none of the peptides as such elicited a reactogenic response in the guinea pig model, no peptides were eliminated from further consideration by this screen. To minimize chances for reactogenicity of the epitope vaccine in its final formulation, one delivery strategy would be to use adenoviral vectors delivered intramuscularly. In this way one could avoid the use of adjuvants that cause localized inflammation at the injection site; such vectors have had a good safety profile in human clinical trials and have been shown to induce potent CD4 and CD8 T-cell responses (91–93). Reactogenicity of the candidate vaccine in its final formulation should then also include a parallel evaluation of antigenicity in the same animal model as an additional control.

In this study we focused on the identification of antigenic and immunogenic T-cell epitopes and hence the cellular component of the envisaged non-reactogenic multi-epitope vaccine. However, we do not preclude the additional involvement of a significant component of antibody-mediated protection in vaccine design. While studies in murine models using formalin-inactivated *C. burnetii* phase I vaccines (94) and of the human whole cell vaccine Q-VAX® (9) suggest a strong contribution of antibodies to protection, how antibodies could provide efficient protection against this intracellular pathogen is not known. Specificity of the antibody response to Q-VAX® has only been analyzed by immunofluorescence assay against whole cell *C. burnetii*, with the exception of a single study assessing the targets of anti-sera from two Q-VAX® vaccinees by protein microarray (20). As an additional caveat, anti-sera from patients with chronic Q fever recognizing phase I antigens were shown to enhance *C. burnetii* infectivity and replication in macrophages *in vitro* (95). Nevertheless, the presence of *C. burnetii*-specific antibodies after both natural infection and immunization (1, 9, 64), and the protective role of these in animal models (12, 13, 94) warrant the consideration of humoral responses in human vaccine design. In this context, elicitation of epitope-specific antibody responses has been described for a HLA class II multi-epitope DNA vaccine (96). Further evaluation of a candidate multi-epitope vaccine should thus also include assessment of humoral responses. Additionally, vaccine optimization could involve the inclusion of known immunogenic whole proteins (24) or specifically selected sequences for (conformational) B-cell epitopes, including a recently described lipopolysaccharide-targeted peptide mimic (97).

In conclusion, we herein identify for the first time a set of *C. burnetii*-specific HLA class II T-cell epitope clusters that was computationally predicted and shown to bind a broad range of HLA-DR types, elicit immunogenicity in tgHLA-DR3 mice and recalled long-lived memory responses in naturally exposed individuals. Further research will focus on evaluating the immunogenicity and protective efficacy of these desirable candidates for a novel promiscuous multi-epitope-based Q fever vaccine in animal models in an appropriate and non-reactogenic formulation that can be transferred directly into clinical studies in humans.

## Data Availability Statement

All relevant data generated for this study are included in the manuscript and the supplementary files. The raw data supporting the conclusions of this manuscript will be made available by the authors, without undue reservation within the scope allowed by patient privacy regulations, to any qualified researcher.

## Author Contributions

AS, LM, LB, PR, WM, TB, RB, RAB, AG, ADG, AES, and MP conceptualized and designed the study and experiments. Experiments were performed by AS, GR, LM, LB, CB, and AG. Data were analyzed and interpreted by AS, GR, LM, LB, and AES. GR, LM, WM, RAB, AG, ADG, and MP contributed critical reagents, materials, and analytic tools. TB, RB, RAB, AG, ADG, AES, and MP acquired funding and supervised research activities. AS, GR, LM, LB, and AES wrote the manuscript and PR, WM, TB, CB, SR, RB, RAB, AG, ADG, and MP critically reviewed and approved the manuscript.

### Conflict of Interest Statement

AG is a senior officer and shareholder and AS is an employee of Innatoss Laboratories B.V., which provides diagnostic screening for Q fever. ADG and WM are senior officers and shareholders, and LM, GR, and CB are employees of EpiVax, Inc., a company specializing in immunoinformatic analysis. Innatoss Laboratories B.V. and EpiVax, Inc., own patents to technologies utilized by associated authors in the research reported here. The remaining authors declare that the research was conducted in the absence of any commercial or financial relationships that could be construed as a potential conflict of interest.

## References

[1] Eldin C Melenotte C Mediannikov O Ghigo E Million M Edouard S. From Q fever to *Coxiella burnetii* infection: a paradigm change. Clin Microbiol Rev. (2017) 30:115–90. 10.1128/CMR.00045-16 27856520 PMC5217791

[2] Tigertt WD Benenson AS Gochenour WS. Airborne Q fever. Bacteriol Rev. (1961) 25:285–93. 13921201 10.1128/br.25.3.285-293.1961 PMC441106

[3] Madariaga MG Rezai K Trenholme GM Weinstein RA. Q fever: a biological weapon in your backyard. Lancet Infect Dis. (2003) 3:709–21. 10.1016/S1473-3099(03)00804-1 14592601

[4] Kampschreur LM Hagenaars JC Wielders CC Elsman P Lestrade PJ Koning OH. Screening for *Coxiella burnetii* seroprevalence in chronic Q fever high-risk groups reveals the magnitude of the Dutch Q fever outbreak. Epidemiol Infect. (2013) 141:847–51. 10.1017/S0950268812001203 22691867 PMC9151832

[5] Kampschreur LM Oosterheert JJ Hoepelman AI Lestrade PJ Renders NH Elsman P. Prevalence of chronic Q fever in patients with a history of cardiac valve surgery in an area where *Coxiella burnetii* is epidemic. Clin Vaccine Immunol. (2012) 19:1165–9. 10.1128/CVI.00185-12 22695158 PMC3416079

[6] Ruiz S Wolfe DN. Vaccination against Q fever for biodefense and public health indications. Front Microbiol. (2014) 5:726. 10.3389/fmicb.2014.00726 25566235 PMC4267281

[7] Chiu CK Durrheim DN. A review of the efficacy of human Q fever vaccine registered in Australia. N S W Public Health Bull. (2007) 18:133–6. 10.1071/NB07057 17854543

[8] Gefenaite G Munster JM van Houdt R Hak E. Effectiveness of the Q fever vaccine: a meta-analysis. Vaccine (2011) 29:395–8. 10.1016/j.vaccine.2010.11.008 21094268

[9] Marmion BP Ormsbee RA Kyrkou M Wright J Worswick DA Izzo AA. Vaccine prophylaxis of abattoir-associated Q fever: eight years' experience in Australian abattoirs. Epidemiol Infect. (1990) 104:275–87. 10.1017/S0950268800059458 2323360 PMC2271744

[10] Karch CP Burkhard P. Vaccine technologies: from whole organisms to rationally designed protein assemblies. Biochem Pharmacol. (2016) 120:1–14. 10.1016/j.bcp.2016.05.001 27157411 PMC5079805

[11] Zhang G Zhang Y Samuel JE. Components of protective immunity. Adv Exp Med Biol. (2012) 984:91–104. 10.1007/978-94-007-4315-1_5 22711628

[12] Humphres RC Hinrichs DJ. Role of antibody in *Coxiella burnetii* infection. Infect Immun. (1981) 31:641–5. 7216465 10.1128/iai.31.2.641-645.1981 PMC351357

[13] Zhang G Russell-Lodrigue KE Andoh M Zhang Y Hendrix LR Samuel JE. Mechanisms of vaccine-induced protective immunity against *Coxiella burnetii* infection in BALB/c mice. J Immunol. (2007) 179:8372–80. 10.4049/jimmunol.179.12.8372 18056383

[14] Andoh M Zhang G Russell-Lodrigue KE Shive HR Weeks BR Samuel JE. T cells are essential for bacterial clearance, and gamma interferon, tumor necrosis factor alpha, and B cells are crucial for disease development in *Coxiella burnetii* infection in mice. Infect Immun. (2007) 75:3245–55. 10.1128/IAI.01767-06 17438029 PMC1932934

[15] Read AJ Erickson S Harmsen AG. Role of CD4^+^ and CD8^+^ T cells in clearance of primary pulmonary infection with *Coxiella burnetii*. Infect Immun. (2010) 78:3019–26. 10.1128/IAI.00101-10 20351144 PMC2897389

[16] Dellacasagrande J Capo C Raoult D Mege JL. IFN-gamma-mediated control of *Coxiella burnetii* survival in monocytes: the role of cell apoptosis and TNF. J Immunol. (1999) 162:2259–65. 9973502

[17] Ghigo E Capo C Tung CH Raoult D Gorvel JP Mege JL. *Coxiella burnetii* survival in THP-1 monocytes involves the impairment of phagosome maturation: IFN-gamma mediates its restoration and bacterial killing. J Immunol. (2002) 169:4488–95. 10.4049/jimmunol.169.8.4488 12370385

[18] Xiong X Qi Y Jiao J Gong W Duan C Wen B. Exploratory study on Th1 epitope-induced protective immunity against *Coxiella burnetii* infection. PLoS ONE (2014) 9:e87206. 10.1371/journal.pone.0087206 24498044 PMC3907486

[19] Reeves PM Paul SR Sluder AE Brauns TA Poznansky MC. Q-vaxcelerate: a distributed development approach for a new *Coxiella burnetii* vaccine. Hum Vaccine Immunother. (2017) 13:2977–81. 10.1080/21645515.2017.1371377 28933682 PMC5718828

[20] Beare PA Chen C Bouman T Pablo J Unal B Cockrell DC. Candidate antigens for Q fever serodiagnosis revealed by immunoscreening of a *Coxiella burnetii* protein microarray. Clin Vaccine Immunol. (2008) 15:1771–9. 10.1128/CVI.00300-08 18845831 PMC2593175

[21] Chen C Bouman TJ Beare PA Mertens K Zhang GQ Russell-Lodrigue KE. A systematic approach to evaluate humoral and cellular immune responses to *Coxiella burnetii* immunoreactive antigens. Clin Microbiol Infect. (2009) 15 (Suppl. 2):156–7. 10.1111/j.1469-0691.2008.02206.x 19281461 PMC2916703

[22] Chen C Dow C Wang P Sidney J Read A Harmsen A. Identification of CD4^+^ T cell epitopes in C. burnetii antigens targeted by antibody responses. PLoS ONE (2011) 6:e17712. 10.1371/journal.pone.0017712 21423609 PMC3057979

[23] Coleman SA Fischer ER Cockrell DC Voth DE Howe D Mead DJ. Proteome and antigen profiling of *Coxiella burnetii* developmental forms. Infect Immun. (2007) 75:290–8. 10.1128/IAI.00883-06 17088354 PMC1828411

[24] Kowalczewska M Sekeyova Z Raoult D. Proteomics paves the way for Q fever diagnostics. Genome Med. (2011) 3:50. 10.1186/gm266 21801463 PMC3221545

[25] Li Q Niu D Wen B Chen M Qiu L Zhang J. Protective immunity against Q fever induced with a recombinant P1 antigen fused with HspB of *Coxiella burnetii*. Ann N Y Acad Sci. (2005) 1063:130–42. 10.1196/annals.1355.021 16481504

[26] Vigil A Ortega R Nakajima-Sasaki R Pablo J Molina DM Chao CC. Genome-wide profiling of humoral immune response to *Coxiella burnetii* infection by protein microarray. Proteomics (2010) 10:2259–69. 10.1002/pmic.201000064 20391532 PMC2892821

[27] Wang X Xiong X Graves S Stenos J Wen B. Protein array of *Coxiella burnetii* probed with Q fever sera. Sci China Life Sci. (2013) 56:453–9. 10.1007/s11427-013-4472-6 23633077

[28] Wei Y Wang X Xiong X Wen B. *Coxiella burnetii* antigen-stimulated dendritic cells mediated protection against *Coxiella burnetii* in BALB/c mice. J Infect Dis. (2011) 203:283–91. 10.1093/infdis/jiq037 21288829 PMC3071064

[29] Xiong X Wang X Wen B Graves S Stenos J. Potential serodiagnostic markers for Q fever identified in *Coxiella burnetii* by immunoproteomic and protein microarray approaches. BMC Microbiol. (2012) 12:35. 10.1186/1471-2180-12-35 22420424 PMC3386016

[30] Carey KL Newton HJ Luhrmann A Roy CR. The *Coxiella burnetii* Dot/Icm system delivers a unique repertoire of type IV effectors into host cells and is required for intracellular replication. PLoS Pathog. (2011) 7:e1002056. 10.1371/journal.ppat.1002056 21637816 PMC3102713

[31] Chen C Banga S Mertens K Weber MM Gorbaslieva I Tan Y. Large-scale identification and translocation of type IV secretion substrates by *Coxiella burnetii*. Proc Natl Acad Sci USA. (2010) 107:21755–60. 10.1073/pnas.1010485107 21098666 PMC3003115

[32] Luhrmann A Nogueira CV Carey KL Roy CR. Inhibition of pathogen-induced apoptosis by a *Coxiella burnetii* type IV effector protein. Proc Natl Acad Sci USA. (2010) 107:18997–9001. 10.1073/pnas.1004380107 20944063 PMC2973885

[33] Sekeyova Z Kowalczewska M Vincentelli R Decloquement P Flores-Ramirez G Skultety L. Characterization of antigens for Q fever serodiagnostics. Acta Virol. (2010) 54:173–80. 10.4149/av_2010_03_173 20822309

[34] van Schaik EJ Chen C Mertens K Weber MM Samuel JE Molecular pathogenesis of the obligate intracellular bacterium *Coxiella burnetii*. Nat Rev Microbiol. (2013) 11:561–73. 10.1038/nrmicro3049 23797173 PMC4134018

[35] Voth DE Beare PA Howe D Sharma UM Samoilis G Cockrell DC. The *Coxiella burnetii* cryptic plasmid is enriched in genes encoding type IV secretion system substrates. J Bacteriol. (2011) 193:1493–503. 10.1128/JB.01359-10 21216993 PMC3067651

[36] Voth DE Howe D Beare PA Vogel JP Unsworth N Samuel JE. The *Coxiella burnetii* ankyrin repeat domain-containing protein family is heterogeneous, with C-terminal truncations that influence Dot/Icm-mediated secretion. J Bacteriol. (2009) 191:4232–42. 10.1128/JB.01656-08 19411324 PMC2698476

[37] Weber MM Chen C Rowin K Mertens K Galvan G Zhi H. Identification of *Coxiella burnetii* type IV secretion substrates required for intracellular replication and *Coxiella*-containing vacuole formation. J Bacteriol. (2013) 195:3914–24. 10.1128/JB.00071-13 23813730 PMC3754607

[38] UniProt Consortium T UniProt: the universal protein knowledgebase. Nucleic Acids Res. (2018) 46:2699 10.1093/nar/gky092 29425356 PMC5861450

[39] Kuley R Smith HE Janse I Harders FL Baas F Schijlen E. First complete genome sequence of the Dutch veterinary *Coxiella burnetii* strain NL3262, originating from the largest global Q fever outbreak, and draft genome sequence of its epidemiologically linked chronic human isolate NLhu3345937. Genome Announc. (2016) 4:e00245–16. 10.1128/genomeA.00245-16 27103714 PMC4841129

[40] Moise L Gutierrez A Kibria F Martin R Tassone R Liu R. iVAX: an integrated toolkit for the selection and optimization of antigens and the design of epitope-driven vaccines. Hum Vaccine Immunother. (2015) 11:2312–21. 10.1080/21645515.2015.1061159 26155959 PMC4635942

[41] Sette A Sidney J. Nine major HLA class I supertypes account for the vast preponderance of HLA-A and -B polymorphism. Immunogenetics (1999) 50:201–12. 10.1007/s002510050594 10602880

[42] Southwood S Sidney J Kondo A del Guercio MF Appella E Hoffman S. Several common HLA-DR types share largely overlapping peptide binding repertoires. J Immunol. (1998) 160:3363–73. 9531296

[43] De Groot AS Martin W Reducing risk, improving outcomes: bioengineering less immunogenic protein therapeutics. Clin Immunol. (2009) 131:189–201. 10.1016/j.clim.2009.01.009 19269256

[44] Moise L Gutierrez AH Bailey-Kellogg C Terry F Leng Q Abdel Hady KM. The two-faced T cell epitope: examining the host-microbe interface with JanusMatrix. Hum Vaccine Immunother. (2013) 9:1577–86. 10.4161/hv.24615 23584251 PMC3974887

[45] Steere AC Klitz W Drouin EE Falk BA Kwok WW Nepom GT. Antibiotic-refractory Lyme arthritis is associated with HLA-DR molecules that bind a *Borrelia burgdorferi* peptide. J Exp Med. (2006) 203:961–71. 10.1084/jem.20052471 16585267 PMC3212725

[46] Buchli R VanGundy RS Hickman-Miller HD Giberson CF Bardet W Hildebrand WH. Real-time measurement of *in vitro* peptide binding to soluble HLA-A^*^0201 by fluorescence polarization. Biochemistry (2004) 43:14852–63. 10.1021/bi048580q 15544356

[47] Mangalam AK Khare M Krco C Rodriguez M David C. Identification of T cell epitopes on human proteolipid protein and induction of experimental autoimmune encephalomyelitis in HLA class II-transgenic mice. Eur J Immunol. (2004) 34:280–90. 10.1002/eji.200324597 14971054

[48] Morroy G Van Der Hoek W Nanver ZD Schneeberger PM Bleeker-Rovers CP Van Der Velden J. The health status of a village population, 7 years after a major Q fever outbreak. Epidemiol Infect. (2016) 144:1153–62. 10.1017/S0950268815002472 26560803

[49] Karagiannis I Schimmer B Van Lier A Timen A Schneeberger P Van Rotterdam B. Investigation of a Q fever outbreak in a rural area of The Netherlands. Epidemiol Infect. (2009) 137:1283–94. 10.1017/S0950268808001908 19161644

[50] Morroy G van der Hoek W Albers J Coutinho RA Bleeker-Rovers CP Schneeberger PM. Population screening for chronic Q-fever seven years after a major outbreak. PLoS ONE (2015) 10:e0131777. 10.1371/journal.pone.0131777 26132155 PMC4489093

[51] Shiina T Suzuki S Ozaki Y Taira H Kikkawa E Shigenari A. Super high resolution for single molecule-sequence-based typing of classical HLA loci at the 8-digit level using next generation sequencers. Tissue Antigens (2012) 80:305–16. 10.1111/j.1399-0039.2012.01941.x 22861646

[52] Sidney J Peters B Frahm N Brander C Sette A. HLA class I supertypes: a revised and updated classification. BMC Immunol. (2008) 9:1. 10.1186/1471-2172-9-1 18211710 PMC2245908

[53] Calarota SA Baldanti F. Enumeration and characterization of human memory T cells by enzyme-linked immunospot assays. Clin Dev Immunol. (2013) 2013:637649. 10.1155/2013/637649 24319467 PMC3844203

[54] Subbramanian RA Basha S Brady RC Hazenfeld S Shata MT Bernstein DI. Age-related changes in magnitude and diversity of cross-reactive CD4^+^ T-cell responses to the novel pandemic H1N1 influenza hemagglutinin. Hum Immunol. (2010) 71:957–63. 10.1016/j.humimm.2010.07.005 20650295

[55] Baeten LA Podell BK Sluder AE Garritsen A Bowen RA Poznansky MC. Standardized guinea pig model for Q fever vaccine reactogenicity. PLoS ONE (2018) 13:e0205882. 10.1371/journal.pone.0205882 30312355 PMC6185858

[56] Xiong X Meng Y Wang X Qi Y Li J Duan C. Mice immunized with bone marrow-derived dendritic cells stimulated with recombinant *Coxiella burnetii* Com1 and Mip demonstrate enhanced bacterial clearance in association with a Th1 immune response. Vaccine (2012) 30:6809–15. 10.1016/j.vaccine.2012.09.017 23000126

[57] Moise L Tassone R Latimer H Terry F Levitz L Haran JP. Immunization with cross-conserved H1N1 influenza CD4^+^ T-cell epitopes lowers viral burden in HLA DR3 transgenic mice. Hum Vaccine Immunother. (2013) 9:2060–8. 10.4161/hv.26511 24045788 PMC3906390

[58] Moise L Terry F Ardito M Tassone R Latimer H Boyle C. Universal H1N1 influenza vaccine development: identification of consensus class II hemagglutinin and neuraminidase epitopes derived from strains circulating between 1980 and 2011. Hum Vaccine Immunother. (2013) 9:1598–607. 10.4161/hv.25598 23846304

[59] Gonzalez-Galarza FF Takeshita LY Santos EJ Kempson F Maia MH daSilva AL. Allele frequency net 2015 update: new features for HLA epitopes, KIR and disease and HLA adverse drug reaction associations. Nucleic Acids Res. (2015) 43:D784–8. 10.1093/nar/gku1166 25414323 PMC4383964

[60] Schipper RF Schreuder GM D'Amaro J Oudshoorn M. HLA gene and haplotype frequencies in Dutch blood donors. Tissue Antigens (1996) 48:562–74. 10.1111/j.1399-0039.1996.tb02670.x 8988539

[61] Wilhelmsen CL Waag DM. Guinea pig abscess/hypersensitivity model for study of adverse vaccination reactions induced by use of Q fever vaccines. Comp Med. (2000) 50:374–8. 11020154

[62] Izzo AA Marmion BP Worswick DA. Markers of cell-mediated immunity after vaccination with an inactivated, whole-cell Q fever vaccine. J Infect Dis. (1988) 157:781–9. 10.1093/infdis/157.4.781 3346570

[63] Jerrells TR Mallavia LP Hinrichs DJ Detection of long-term cellular immunity to *Coxiella burnetii* as assayed by lymphocyte transformation. Infect Immun. (1975) 11:280–6. 1112616 10.1128/iai.11.2.280-286.1975 PMC415057

[64] Kersh GJ Fitzpatrick KA Self JS Biggerstaff BJ Massung RF. Long-Term immune responses to *Coxiella burnetii* after vaccination. Clin Vaccine Immunol. (2013) 20:129–33. 10.1128/CVI.00613-12 23192629 PMC3571276

[65] Chao CC Chen HW Li X Xu WB Hanson B Ching WM. Identification, cloning, and expression of potential diagnostic markers for Q fever. Ann N Y Acad Sci. (2005) 1063:76–8. 10.1196/annals.1355.010 16481493

[66] Flores-Ramirez G Danchenko M Quevedo-Diaz M Skultety L. Reliable tool for detection of novel *Coxiella burnetii* antigens, using immobilized human polyclonal antibodies. J Chromatogr B Analyt Technol Biomed Life Sci. (2017) 1047:84–91. 10.1016/j.jchromb.2016.08.044 27639449

[67] Gerlach C Skultety L Henning K Neubauer H Mertens K. *Coxiella burnetii* immunogenic proteins as a basis for new Q fever diagnostic and vaccine development. Acta Virol. (2017) 61:377–90. 10.4149/av_2017_320 28854806

[68] Jiao J Xiong X Qi Y Gong W Duan C Yang X. Serological characterization of surface-exposed proteins of *Coxiella burnetii*. Microbiology (2014) 160 (Pt 12):2718–31. 10.1099/mic.0.082131-0 25298245

[69] Papadioti A Markoutsa S Vranakis I Tselentis Y Karas M Psaroulaki A. A proteomic approach to investigate the differential antigenic profile of two *Coxiella burnetii* strains. J Proteomics (2011) 74:1150–9. 10.1016/j.jprot.2011.04.016 21565289

[70] Sekeyova Z Kowalczewska M Decloquement P Pelletier N Spitalska E Raoult D. Identification of protein candidates for the serodiagnosis of Q fever endocarditis by an immunoproteomic approach. Eur J Clin Microbiol Infect Dis. (2009) 28:287–95. 10.1007/s10096-008-0621-4 18797945

[71] Vigil A Chen C Jain A Nakajima-Sasaki R Jasinskas A Pablo J. Profiling the humoral immune response of acute and chronic Q fever by protein microarray. Mol Cell Proteomics (2011) 10:M110.006304. 10.1074/mcp.m110.006304 21817167 PMC3205856

[72] Xiong X Jiao J Gregory AE Wang P Bi Y Wang X. Identification of *Coxiella burnetii* CD8^+^ T-cell epitopes and delivery by attenuated listeria monocytogenes as a vaccine vector in a C57BL/6 mouse model. J Infect Dis. (2017) 215:1580–9. 10.1093/infdis/jiw470 27703037 PMC6281342

[73] Comas I Chakravartti J Small PM Galagan J Niemann S Kremer K. Human T cell epitopes of *Mycobacterium tuberculosis* are evolutionarily hyperconserved. Nat Genet. (2010) 42:498–503. 10.1038/ng.590 20495566 PMC2883744

[74] Ernst JD. Antigenic variation and immune escape in the MTBC. Adv Exp Med Biol. (2017) 1019:171–90. 10.1007/978-3-319-64371-7_9 29116635 PMC5718154

[75] Tientcheu LD Koch A Ndengane M Andoseh G Kampmann B Wilkinson RJ. Immunological consequences of strain variation within the *Mycobacterium tuberculosis* complex. Eur J Immunol. (2017) 47:432–45. 10.1002/eji.201646562 28150302 PMC5363233

[76] Lamonaca V Missale G Urbani S Pilli M Boni C Mori C. Conserved hepatitis C virus sequences are highly immunogenic for CD4^+^ T cells: implications for vaccine development. Hepatology (1999) 30:1088–98. 10.1002/hep.510300435 10498664

[77] Penna A Missale G Lamonaca V Pilli M Mori C Zanelli P. Intrahepatic and circulating HLA class II-restricted, hepatitis C virus-specific T cells: functional characterization in patients with chronic hepatitis C. Hepatology (2002) 35:1225–36. 10.1053/jhep.2002.33153 11981773

[78] Almeida RR Rosa DS Ribeiro SP Santana VC Kallas EG Sidney J. Broad and cross-clade CD4^+^ T-cell responses elicited by a DNA vaccine encoding highly conserved and promiscuous HIV-1 M-group consensus peptides. PLoS ONE (2012) 7:e45267. 10.1371/journal.pone.0045267 23028895 PMC3445454

[79] Fonseca SG Coutinho-Silva A Fonseca LA Segurado AC Moraes SL Rodrigues H. Identification of novel consensus CD4 T-cell epitopes from clade B HIV-1 whole genome that are frequently recognized by HIV-1 infected patients. AIDS (2006) 20:2263–73. 10.1097/01.aids.0000253353.48331.5f 17117012

[80] Izzo AA Marmion BP Variation in interferon-gamma responses to *Coxiella burnetii* antigens with lymphocytes from vaccinated or naturally infected subjects. Clin Exp Immunol. (1993) 94:507–15. 10.1111/j.1365-2249.1993.tb08226.x 8252811 PMC1534438

[81] Buttrum L Ledbetter L Cherla R Zhang Y Mitchell WJ Zhang G. Both major histocompatibility complex class I (MHC-I) and MHC-II molecules are required, while MHC-I appears to play a critical role in host defense against primary *Coxiella burnetii* infection. Infect Immun. (2018) 86:e00602–17. 10.1128/IAI.00602-17 29311245 PMC5865044

[82] Li HM Hiroi T Zhang Y Shi A Chen G De S. TCRbeta repertoire of CD4^+^ and CD8^+^ T cells is distinct in richness, distribution, and CDR3 amino acid composition. J Leukoc Biol. (2016) 99:505–13. 10.1189/jlb.6A0215-071RR 26394815 PMC5338248

[83] Amara RR Nigam P Sharma S Liu J Bostik V. Long-lived poxvirus immunity, robust CD4 help, and better persistence of CD4 than CD8 T cells. J Virol. (2004) 78:3811–6. 10.1128/JVI.78.8.3811-3816.2004 15047796 PMC374286

[84] Hammarlund E Lewis MW Hanifin JM Mori M Koudelka CW Slifka MK. Antiviral immunity following smallpox virus infection: a case-control study. J Virol. (2010) 84:12754–60. 10.1128/JVI.01763-10 20926574 PMC3004327

[85] Nyendak MR Park B Null MD Baseke J Swarbrick G Mayanja-Kizza H. *Mycobacterium tuberculosis* specific CD8^+^ T cells rapidly decline with antituberculosis treatment. PLoS ONE (2013) 8:e81564. 10.1371/journal.pone.0081564 24324704 PMC3852504

[86] Axelsson-Robertson R Rao M Loxton AG Walzl G Bates M Zumla A. Frequency of *Mycobacterium tuberculosis*-specific CD8^+^ T-cells in the course of anti-tuberculosis treatment. Int J Infect Dis. (2015) 32:23–9. 10.1016/j.ijid.2015.01.017 25809751

[87] Pearce EL Shedlock DJ Shen H. Functional characterization of MHC class II-restricted CD8^+^CD4^−^ and CD8^−^CD4^−^ T cell responses to infection in CD4^−/−^ mice. J Immunol. (2004) 173:2494–9. 10.4049/jimmunol.173.4.2494 15294964

[88] Ranasinghe S Lamothe PA Soghoian DZ Kazer SW Cole MB Shalek AK. Antiviral CD8^+^ T cells restricted by human leukocyte antigen class II exist during natural HIV infection and exhibit clonal expansion. Immunity (2016) 45:917–30. 10.1016/j.immuni.2016.09.015 27760342 PMC5077698

[89] Kampschreur LM Wegdam-Blans MC Wever PC Renders NH Delsing CE Sprong T. Chronic Q fever diagnosis- consensus guideline versus expert opinion. Emerg Infect Dis. (2015) 21:1183–8. 10.3201/eid2107.130955 26277798 PMC4480373

[90] Chentoufi AA Kritzer E Yu DM Nesburn AB Benmohamed L. Towards a rational design of an asymptomatic clinical herpes vaccine: the old, the new, and the unknown. Clin Dev Immunol. (2012) 2012:187585. 10.1155/2012/187585 22548113 PMC3324142

[91] Gilbert SC. T-cell-inducing vaccines—what's the future. Immunology (2012) 135:19–26. 10.1111/j.1365-2567.2011.03517.x 22044118 PMC3246649

[92] Humphreys IR Sebastian S. Novel viral vectors in infectious diseases. Immunology (2018) 153:1–9. 10.1111/imm.12829 28869761 PMC5721250

[93] Zhang C Zhou D. Adenoviral vector-based strategies against infectious disease and cancer. Hum Vaccine Immunother. (2016) 12:2064–74. 10.1080/21645515.2016.1165908 27105067 PMC4994731

[94] Zhang G Peng Y Schoenlaub L Elliott A Mitchell W Zhang Y. Formalin-inactivated *Coxiella burnetii* phase I vaccine-induced protection depends on B cells to produce protective IgM and IgG. Infect Immun. (2013) 81:2112–22. 10.1128/IAI.00297-13 23545296 PMC3676018

[95] Desnues B Imbert G Raoult D Mege JL Ghigo E. Role of specific antibodies in *Coxiella burnetii* infection of macrophages. Clin Microbiol Infect. (2009) 15 (Suppl. 2):161–2. 10.1111/j.1469-0691.2008.02208.x 19281459

[96] Bounds CE Terry FE Moise L Hannaman D Martin WD De Groot AS. An immunoinformatics-derived DNA vaccine encoding human class II T cell epitopes of Ebola virus, Sudan virus, and Venezuelan equine encephalitis virus is immunogenic in HLA transgenic mice. Hum Vaccine Immunother. (2017) 13:2824–36. 10.1080/21645515.2017.1329788 28575582 PMC5718811

[97] Peng Y Zhang Y Mitchell WJ Zhang G. Development of a lipopolysaccharide-targeted peptide mimic vaccine against Q fever. J Immunol. (2012) 189:4909–20. 10.4049/jimmunol.1201622 23053512 PMC3833726

