# Supplementary material for: Promiscuous Coxiella burnetii CD4 Epitope Clusters Associated With Human Recall Responses Are Candidates for a Novel T-Cell Targeted Multi-Epitope Q Fever Vaccine
Source: Front Immunol. 2019 Feb 15;10:207. doi: 10.3389/fimmu.2019.00207 (PMC6384241; doi:10.3389/fimmu.2019.00207)
Supplement: Supplementary file 1 [file Data_Sheet_1.pdf]

Supplementary Material

Promiscuous *Coxiella burnetii* CD4 Epitope Clusters Associated with Human Recall Responses Are Candidates for a Novel T-cell Targeted Multi-Epitope Q fever Vaccine

Authors

Anja Scholzen, Guilhem Richard, Leonard Moise, Laurie A. Baeten, Patrick M. Reeves, William D. Martin, Timothy A. Brauns, Christine M. Boyle, Susan Raju Paul, Richard Bucala, Richard A. Bowen, Anja Garritsen, Anne S. De Groot, Ann E. Sluder*, Mark C. Poznansky

*** Correspondence:** Dr. Ann E Sluder asluder@mgh.harvard.edu

Table S1. Accuracy of T-cell epitope predictions determined in HLA binding assays

HLA Class	Allele	Total	True positive prediction[3]	False positive prediction[4]	True negative prediction[5]	False negative prediction[6]	Positive predictive accuracy
**Class II[1]**	**DRB1*0101**	50	43	3	1	3	88%
	**DRB1*0301**	50	21	16	9	4	60%
	**DRB1*0401**	50	33	13	1	3	68%
	**DRB1*0701**	50	40	3	1	6	82%
	**DRB1*0801**	50	25	15	7	3	64%
	**DRB1*1101**	50	39	6	1	4	80%
	**DRB1*1301**	50	34	6	3	7	74%
	**DRB1*1501**	50	43	4	0	3	86%
	**Total**	**400**	**278**	**66**	**23**	**33**	**75%**
**Class I[2]**	**A*0101**	11	8	3	-	-	73%
	**A*0201**	11	11	0	-	-	100%
	**A*0301**	10	10	0	-	-	100%
	**A*2402**	11	11	0	-	-	100%
	**B*0702**	11	8	3	-	-	73%
	**B*4403**	11	8	3	-	-	73%
	**Total**	**65**	**56**	**6**	**-**	**-**	**86%**

[1] HLA class II peptides were selected for broad reactivity and assayed for binding to all class II alleles available regardless of positive or negative prediction
[2] HLA class I peptides were assayed for binding only to the primary allele they were predicted to bind
[3] Bioinformatic prediction was confirmed by *in vitro* binding
[4] Bioinformatic prediction was not confirmed by *in vitro* binding
[5] Binding was neither predicted nor observed *in vitro*
[6] *In vitro* binding was observed despite negative bioinformatic prediction

Table S2. Human donor selection for HLA class II T-cell epitope antigenicity screening

	Total cohort				Subcohort for HLA class II T-cell epitope antigenicity screening			Expected frequency[3]
**Group**	**Total cohort**	**A (contr.)**	**B (asympt.)**	**C (sympt.)**	**A (contr.)**	**B (asympt.)**	**C (sympt.)**	
**N**	**136**	**26**	**73**	**37**	**21**	**33**	**23**	
**Coxiella-specific IFNγ response in pg/ml (median, IQR)[1]**		3 [1-10.3]	330 [168-660]	348 [180-717]	3 [1-8]	460 [214-699]	434 [312-988]	
**HLA-DR1[2]**	28 (20.6%)	8 (30.8%)	12 (16.4%)	8 (21.6%)	7 (33.3%)	6 (18.2%)	5 (21.7%)	12.2 – 19.8%
**HLA-DR3**	29 (21.3%)	5 (19.2%)	19 (26.0%)	5 (13.5%)	5 (23.8%)	8 (24.2%)	5 (21.7%)	12.9 – 25.0%
**HLA-DR4**	39 (28.7%)	4 (15.4%)	25 (34.2%)	10 (27.0%)	3 (14.3%)	9 (27.3%)	6 (26.1%)	15.1 – 28.3%
**HLA-DR7**	31 (22.8%)	4 (15.4%)	15 (20.5%)	12 (32.4%)	4 (19%)	9 (27.3%)	6 (26.1%)	11.2 – 26.2%
**HLA-DR8**	8 (5.9%)	2 (7.7%)	6 (8.2%)	0 (0.0%)	2 (0.9%)	4 (12.1%)	0 (0.0%)	3.9 – 5.5%
**HLA-DR11**	22 (16.2%)	4 (15.4%)	13 (17.8%)	5 (13.5%)	4 (19%)	7 (21.1%)	5 (21.7%)	11.3 – 17.0%
**HLA-DR13**	41 (30.1%)	8 (30.8%)	20 (27.4%)	13 (35.1%)	7 (33.3%)	5 (15.2%)	7 (30.4%)	12.0 – 28.4%
**HLA-DR15**	35 (25.7%)	9 (34.6%)	19 (26.0%)	7 (18.9%)	5 (23.8%)	9 (27.3%)	5 (21.7%)	8.0 – 25.5%

[1] At inclusion into the study in October 2015, medium only background subtracted

[2] Frequencies of subjects expressing a copy of the indicated HLA allele within each group. Donors that were homozygous for a single allele are counted once. Shown as total N per group and (%)

[3] Range of HLA frequencies reported in the Dutch population by (i) allelefrequencies.net, combination of the "Germany DKMS – Netherland minority", "Netherlands Leiden", and "Netherlands UMCU" populations, (ii) by Schipper et al. [1] and (iii) by Southwood et al. [2], (Caucasian population, HLA class II only)

Table S3. Human donor selection for HLA class I T-cell epitope antigenicity screening

	Total cohort				Subcohort for HLA class I T-cell epitope antigenicity screening			Expected frequency[3]
**Group**	**Total cohort**	**A (contr.)**	**B (asympt.)**	**C (sympt.)**	**A (contr.)**	**B (asympt.)**	**C (sympt.)**	
**N**	**136**	**26**	**73**	**37**	**20**	**32**	**25**	
**Coxiella-specific IFNγ response in pg/ml (median, IQR)[1]**		3 [1-10.3]	330 [168-660]	348 [180-717]	3.5 [1.3-10.5]	441 [203-699]	378 [212-949]	
**HLA-A1[2]**	52 (38.2%)	6 (23.1%)	31 (42.5%)	15 (40.5%)	5 (25%)	11 (34.3%)	9 (36%)	24.3 – 44.6%
**HLA-A2**	66 (48.5%)	15 (57.7%)	33 (45.2%)	18 (48.6%)	10 (50%)	13 (40.6%)	13 (52%)	30.2 – 52.6%
**HLA-A3**	56 (41.2%)	12 (46.2%)	32 (43.8%)	12 (32.4%)	9 (45%)	14 (43.8%)	8 (32%)	21.1 – 38.7%
**HLA-A11**	18 (13.2%)	6 (23.1%)	8 (11.0%)	4 (10.8%)	4 (20%)	4 (12.5%)	3 (12%)	5.2 – 11.6%
**HLA-A24**	29 (21.3%)	5 (19.2%)	14 (19.2%)	10 (27.0%)	5 (25%)	12 (37.5%)	10 (40%)	11.1 – 19.1%
**HLA-A68**	25 (18.4%)	4 (15.4%)	15 (20.5%)	6 (16.2%)	3 (15%)	6 (18.8%)	4 (16%)	7.2 – 14.9%
**HLA-B7**	58 (42.6%)	13 (50.0%)	27 (37.0%)	18 (48.6%)	8 (40%)	15 (46.9%)	12 (48%)	18.4 – 37.1%
**HLA-B8**	29 (21.3%)	5 (19.2%)	18 (24.7%)	6 (16.2%)	5 (25%)	7 (21.9%)	6 (24%)	12.6 – 22.7%
**HLA-B27**	23 (16.9%)	4 (15.4%)	16 (21.9%)	3 (8.1%)	3 (15%)	9 (28.1%)	3 (12%)	5.4 – 13.9%
**HLA-B35**	45 (33.1%)	9 (34.6%)	21 (28.8%)	15 (40.5%)	7 (35%)	10 (31.3%)	9 (36%)	17.9 – 33.5%
**HLA-B44**	72 (52.9%)	13 (50.0%)	40 (54.8%)	19 (51.4%)	10 (50%)	13 (40.6%)	11 (44%)	31.3 – 57.2%

[1] At inclusion into the study in October 2015, medium only background subtracted

[2] Frequencies of subjects expressing a copy of the indicated HLA allele within each group. Donors that were homozygous for a single allele are counted once. Shown as total N per group and (%)

[3] Range of HLA frequencies reported in the Dutch population by (i) allelefrequencies.net, combination of the "Germany DKMS – Netherland minority", "Netherlands Leiden", and "Netherlands UMCU" populations, (ii) by Schipper et al. [1] and (iii) by Southwood et al. [2], (Caucasian population, HLA class II only)

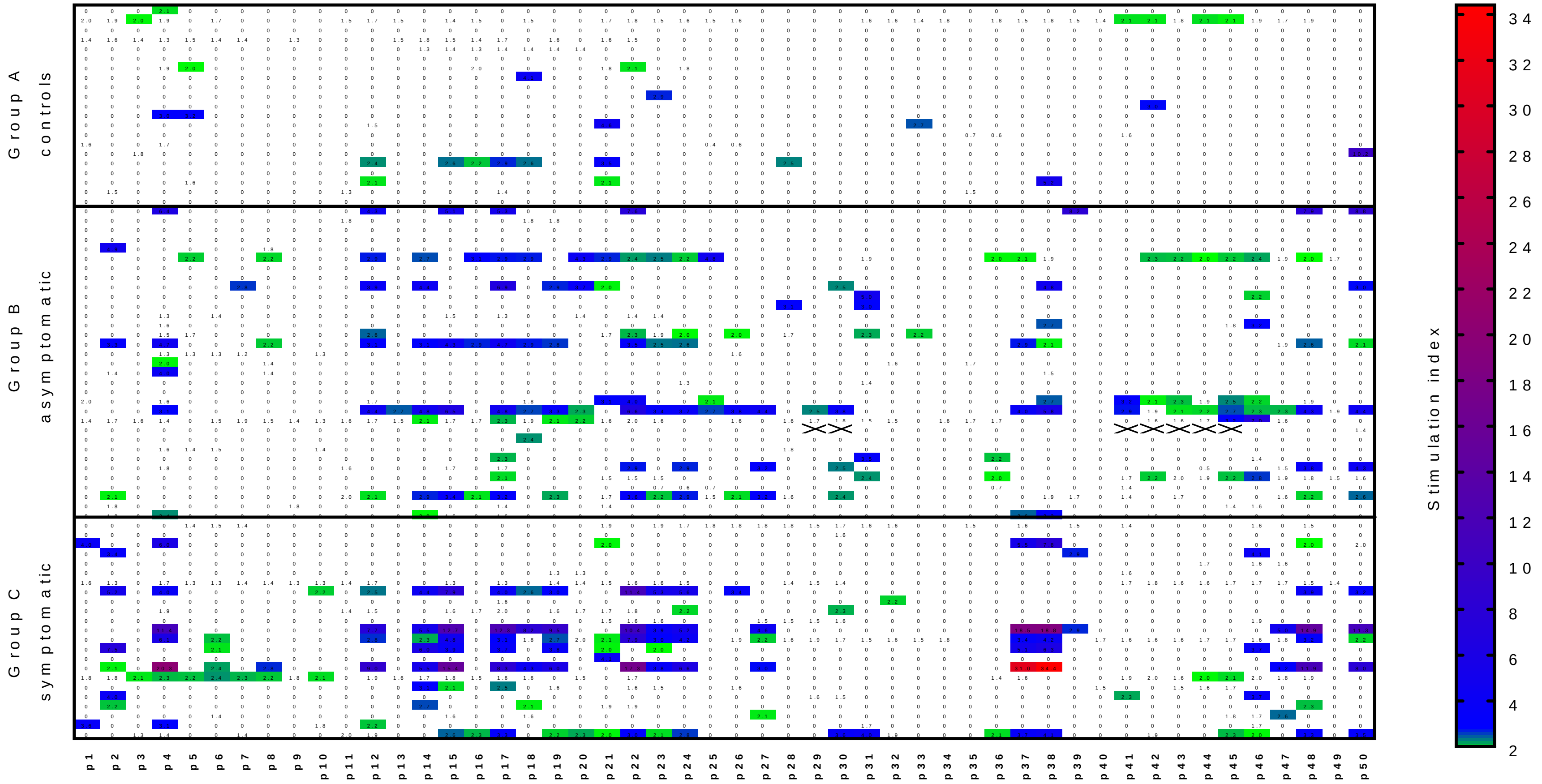

Figure S1. Overview of human IFNγ responses to HLA class II peptides. Individual IFNγ responses to HLA class II peptides determined by cultured ELISpot are depicted as stimulation indices (SI) for all donors from group A (n=21), B (n=33) and C (n=23). Each row shows data from one donor, each column responses to one of the 50 class II peptides. Responses not significantly different from background and/or lower than an average of 10 spots/well are denoted as 0. Significant responses with a SI≥2 are color coded as per heatmap legend. Crosses indicate conditions for which no data are available due to technical error or insufficient cell numbers.

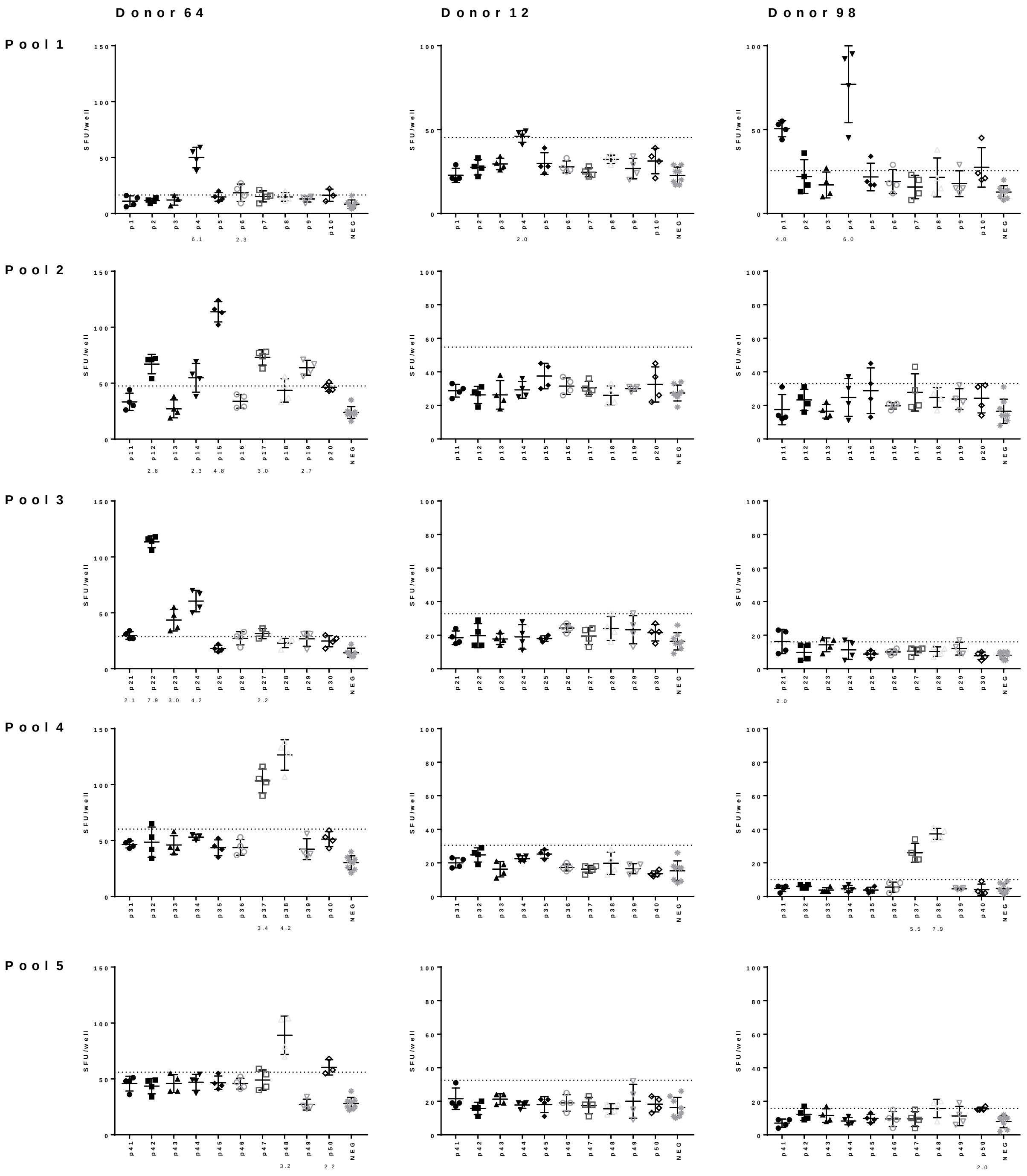

Figure S2. Representative human IFNγ cultured ELISpot responses to HLA class II peptides. HLA class II peptide specific IFNγ responses are shown as absolute spot forming units (SFU) per well for three individual donors. Data are shown per peptide pool expansion culture. Dotted lines indicate the cut-off for positivity, namely a stimulation index (SI) of 2 in reference to medium-only wells (negative control, NEG) per expansion culture, or 10 SFU/well if SI=2 would otherwise be reached at a lower spot count. Positive responses further needed to be significantly higher than NEG wells by one-way ANOVA with Holm- Šídák multiple comparisons post-hoc test. SI values for positive responses are denoted underneath the respective peptide label on the x-axis.

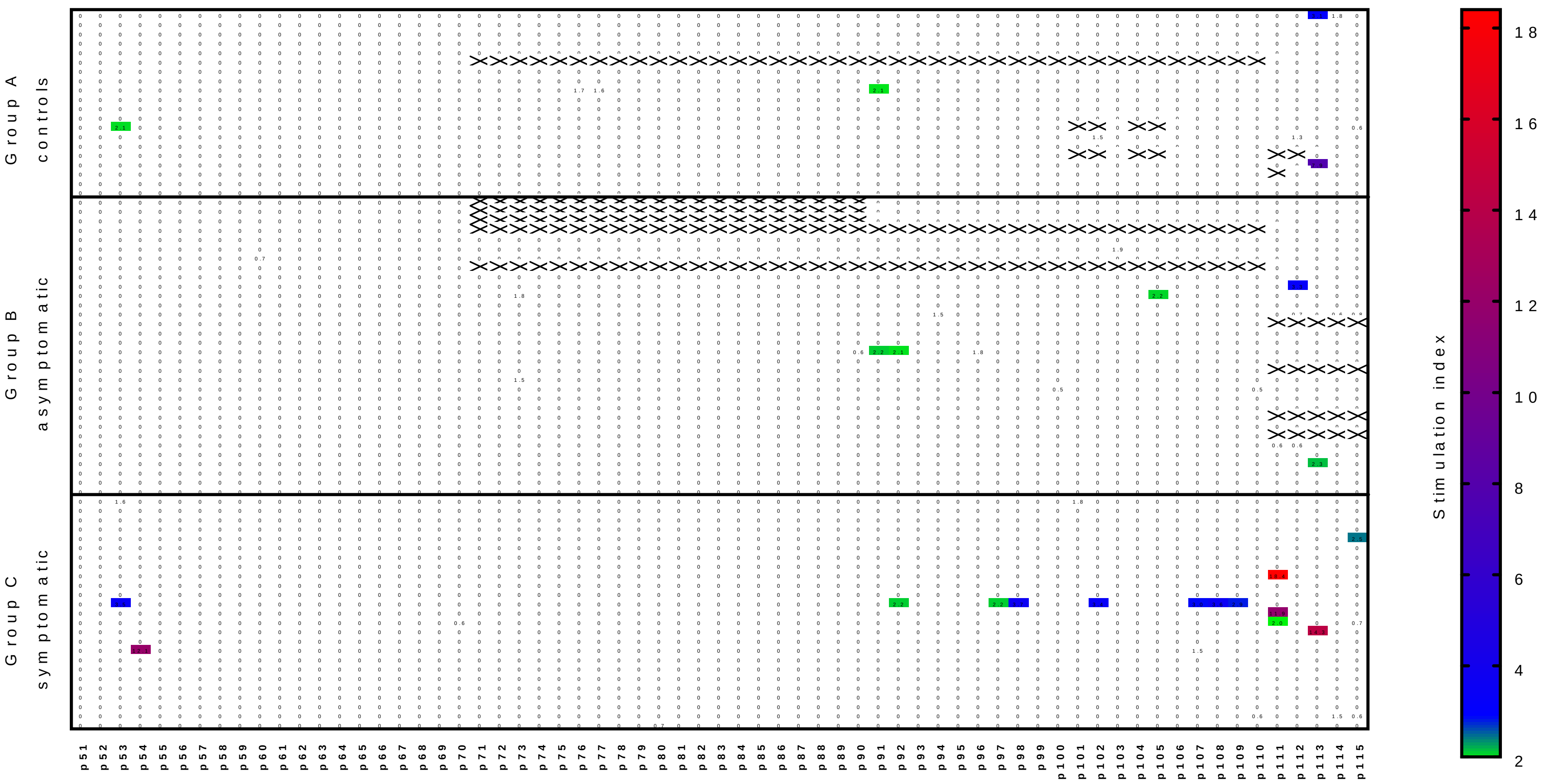

Figure S3. Overview of human IFNγ responses to HLA class I peptides. Individual IFNγ responses to HLA class I peptides determined by cultured ELISpot are depicted as stimulation indices (SI) for all donors from group A (n=20), B (n=32) and C (n=25). Each row shows data from one donor, each column responses to one of the 65 class I peptides. Responses not significantly different from background and/or lower than an average of 10 spots/well are denoted as 0. Significant responses with a SI≥2 are color coded as per heatmap legend. Crosses indicate conditions for which no data are available due to technical error or insufficient cell numbers.

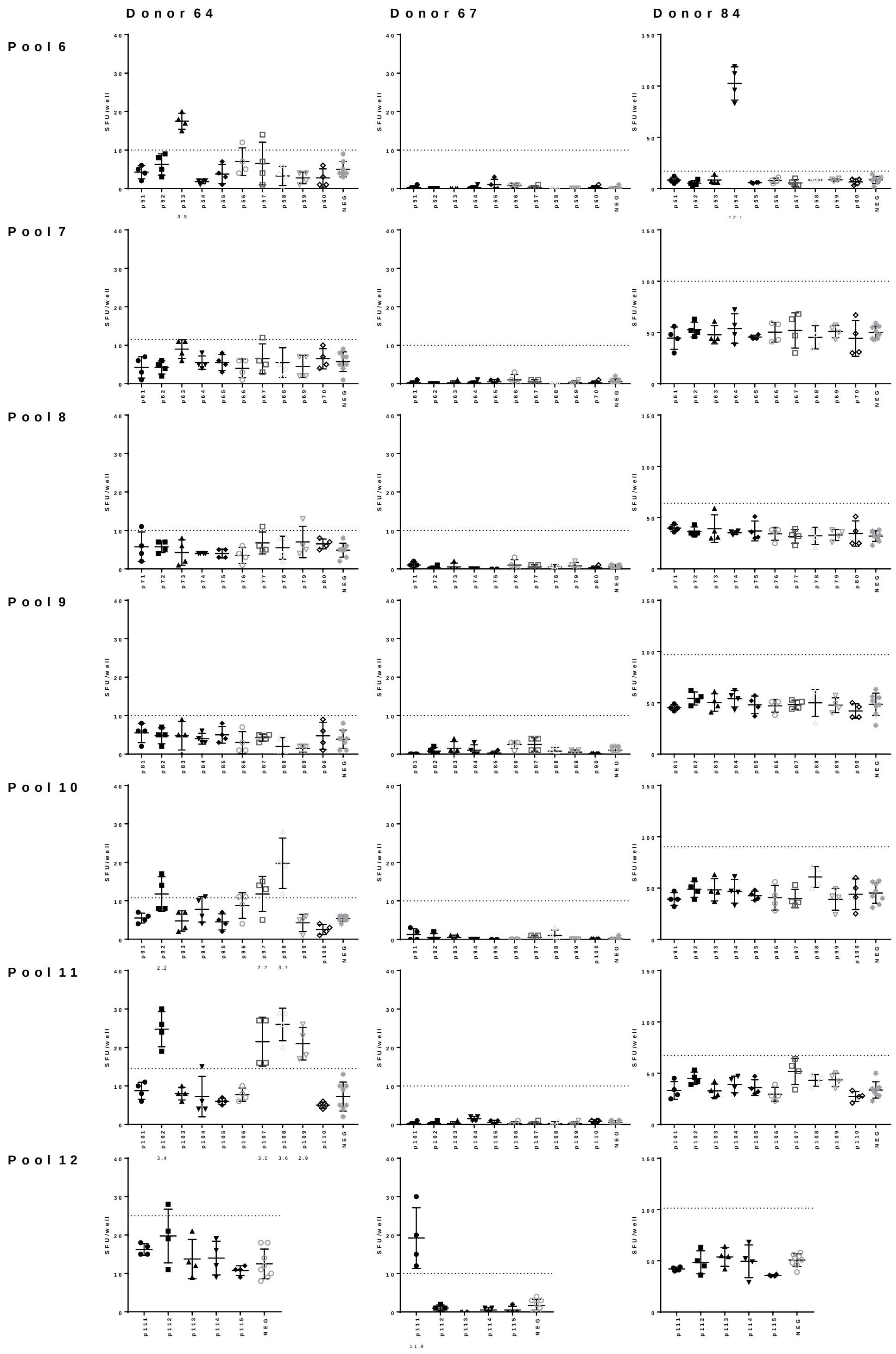

Figure S4. Exemplary human IFNγ cultured ELISpot responses to HLA class I peptides. HLA class I peptide specific IFNγ responses are shown as absolute spot forming units (SFU) per well for three individual donors. Data are shown per peptide pool expansion culture. Dotted lines indicate the cut-off for positivity, namely a stimulation index (SI) of 2 in reference to medium-only wells (negative control, NEG) per expansion culture, or 10 SFU/well if SI=2 would otherwise be reached at a lower spot count. Positive responses further needed to be significantly higher than NEG wells by one-way ANOVA with Holm- Šídák multiple comparisons post-hoc test. SI values for positive responses are denoted underneath the respective peptide label on the x-axis.

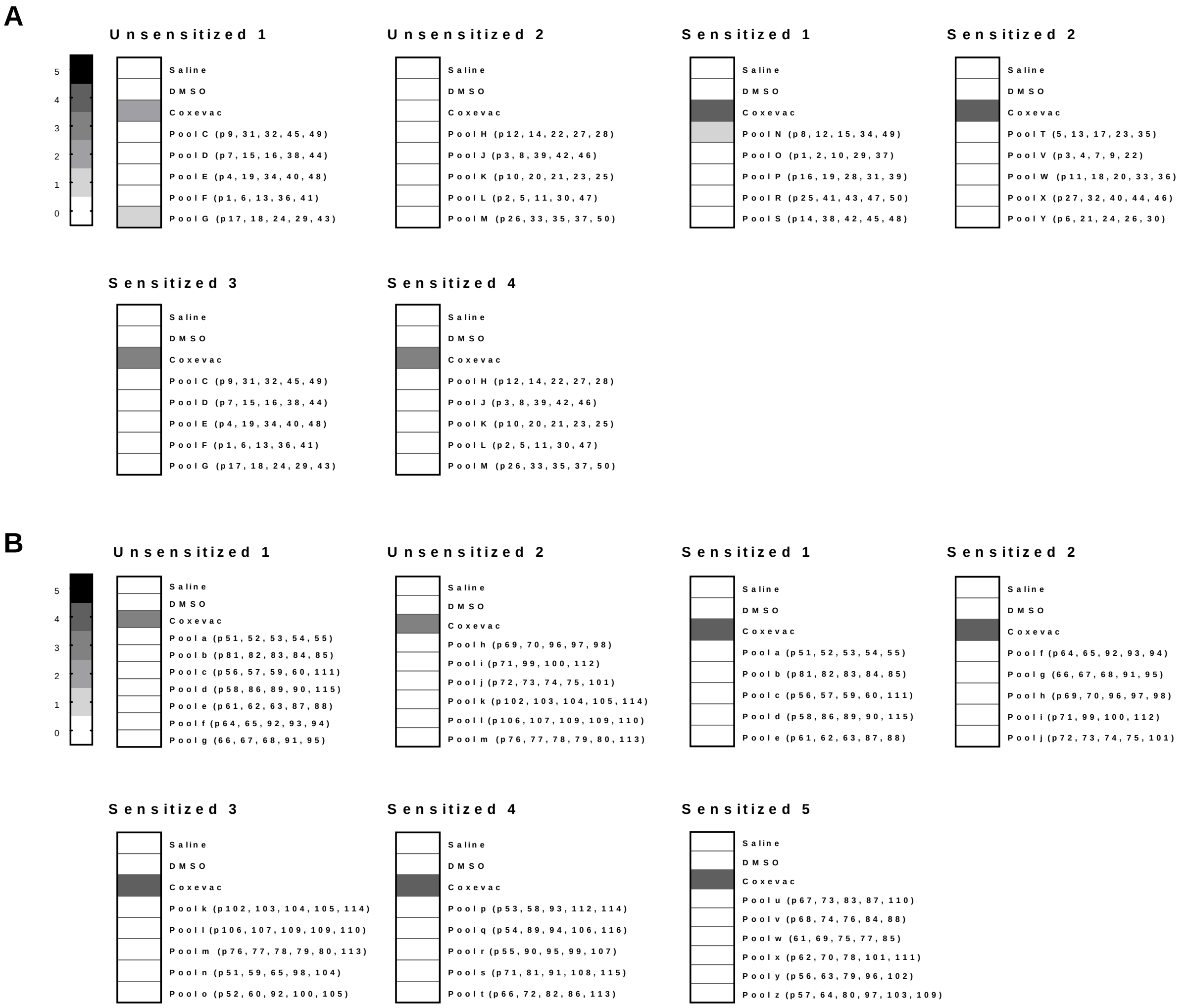

Figure S5: Reactogenicity screening of HLA Class II and I peptides in guinea pigs. Histology scores of guinea pigs challenged intradermally with pools of peptides were assessed separately for HLA class II (A) and class I peptides (B). Each peptide was tested once in unsensitized animals, and twice (in two different pool compositions) in sensitized animals, 42 days after intranasal inoculation with 10^6 *C. burnetii* Nine Mile. Histological scores from skin biopsies collected at day 7 post challenge are represented as gray scale.

Supplementary References

1. Schipper RF, Schreuder GM, D'Amaro J, Oudshoorn M. HLA gene and haplotype frequencies in Dutch blood donors. Tissue Antigens. 1996;48(5):562-74. PubMed PMID: 8988539.
2. Southwood S, Sidney J, Kondo A, del Guercio MF, Appella E, Hoffman S, et al. Several common HLA-DR types share largely overlapping peptide binding repertoires. J Immunol. 1998;160(7):3363-73. PubMed PMID: 9531296.
